# Landscape Epidemiology and Control of Pathogens with Cryptic and Long-Distance Dispersal: Sudden Oak Death in Northern Californian Forests

**DOI:** 10.1371/journal.pcbi.1002328

**Published:** 2012-01-05

**Authors:** João A. N. Filipe, Richard C. Cobb, Ross K. Meentemeyer, Christopher A. Lee, Yana S. Valachovic, Alex R. Cook, David M. Rizzo, Christopher A. Gilligan

**Affiliations:** 1 Department of Plant Sciences, University of Cambridge, Cambridge, United Kingdom; 2 Department of Plant Pathology, University of California-Davis, Davis, California, United States of America; 3 Department of Geography and Earth Sciences, University of North Carolina, Charlotte, North Carolina, United States of America; 4 University of California Cooperative Extension, Eureka, California, United States of America; 5 Statistics and Applied Probability, National University of Singapore, Singapore; Johns Hopkins Bloomberg School of Public Health, United States of America

## Abstract

Exotic pathogens and pests threaten ecosystem service, biodiversity, and crop security globally. If an invasive agent can disperse asymptomatically over long distances, multiple spatial and temporal scales interplay, making identification of effective strategies to regulate, monitor, and control disease extremely difficult. The management of outbreaks is also challenged by limited data on the actual area infested and the dynamics of spatial spread, due to financial, technological, or social constraints. We examine principles of landscape epidemiology important in designing policy to prevent or slow invasion by such organisms, and use *Phytophthora ramorum*, the cause of sudden oak death, to illustrate how shortfalls in their understanding can render management applications inappropriate. This pathogen has invaded forests in coastal California, USA, and an isolated but fast-growing epidemic focus in northern California (Humboldt County) has the potential for extensive spread. The risk of spread is enhanced by the pathogen's generalist nature and survival. Additionally, the extent of cryptic infection is unknown due to limited surveying resources and access to private land. Here, we use an epidemiological model for transmission in heterogeneous landscapes and Bayesian Markov-chain-Monte-Carlo inference to estimate dispersal and life-cycle parameters of *P. ramorum* and forecast the distribution of infection and speed of the epidemic front in Humboldt County. We assess the viability of management options for containing the pathogen's northern spread and local impacts. Implementing a stand-alone host-free “barrier” had limited efficacy due to long-distance dispersal, but combining curative with preventive treatments ahead of the front reduced local damage and contained spread. While the large size of this focus makes effective control expensive, early synchronous treatment in newly-identified disease foci should be more cost-effective. We show how the successful management of forest ecosystems depends on estimating the spatial scales of invasion and treatment of pathogens and pests with cryptic long-distance dispersal.

## Author Summary

We discuss principles governing the spread and management of diseases in natural forest ecosystems. Invasive organisms are damaging world forests and agricultural crops at an increasing rate and severity due to global trade and environmental disturbances. While prevention is the best option, practitioners must decide whether and how to act once a pathogen emerges in a new environment. But, how do we know that an invasion is occurring, its current extent, and its future spread? Emerging pathogens are often observed too late because they are unknown or difficult to detect before causing damage. Once we detect the invader, what can we do to manage and hopefully eliminate it? As many invasions occur rapidly on large geographic scales, small-scale affordable experimentation is not an option, so we need predictive models to gain insight into these questions. Invaders are more challenging if they disperse cryptically and over long distances. We study the case of sudden oak death in California, estimating where and how fast it is spreading, and showing that resources for management must be deployed rationally and early in order to succeed. A promising strategy is curative treatment at the core with preventive protection stretching far around the focus of the outbreak.

## Introduction

**Figure 1:**
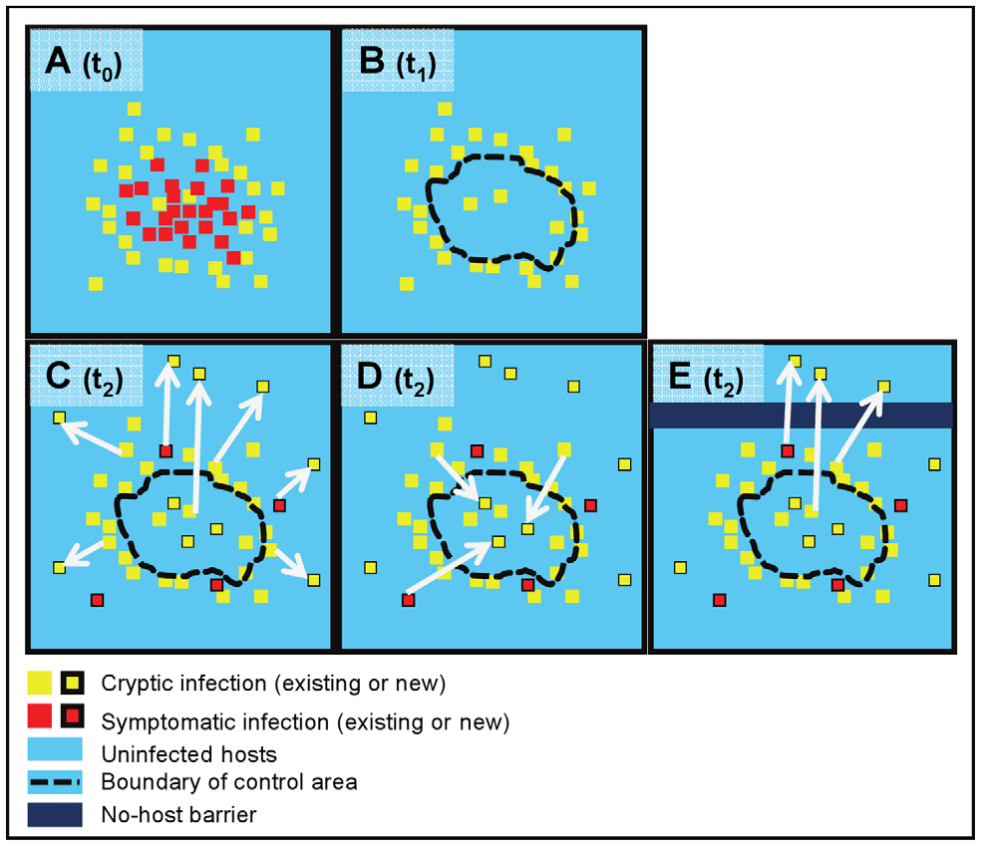
Epidemiological principles in landscape control of plant pathogens with cryptic infection and long-distance dispersal. Rational management (eradication or suppression) of invading pathogens on a heterogeneous landscape requires estimating the extent (including the front) of the cryptic epidemic which is larger than what the prevalence of symptoms suggests at given time t_0_ (**A**). Without this information, treatment (of symptomatic or of all hosts at later time t_1_) is restricted to a control area defined by the observed symptoms, which misses out cryptic infections around (and possibly within) the core of the outbreak (**B**). The degree of mismatch between scales of control and infection depend on the degrees of cryptic and long-distance spread in the pathosystem. At a subsequent time, t_2_, the cryptic infections (some of which have become symptomatic) have continued to spread beyond the control area, expanding the epidemic focus (**C**), and spreading back into the control area if it still contains non-infected hosts (**D**), regardless of the amount of control effort. A barrier treatment (total removal of hosts) ahead of the epidemic front, whether or not combined with treatment of symptoms at the epidemic core, is likely to fail to contain (although it might delay) the outbreak when the pathogen is able to disperse over distances larger than the width of the barrier (**E**). A central concept in invasion is that of *local basic reproduction number* (R_0_), the average number of units infected by a *local* unit at site *x* in an otherwise susceptible landscape. On average, an epidemic occurs at *x*, after inoculation, if R_0_>1(**A**), otherwise transmission is not sustained. Treatment might reduce R_0_ below 1 within the control area (**B**) but not in the rest of the landscape, to where and within where inoculum continues to spread and establish (**C, E**), and from where it is able to re-invade the control area regardless of the local reduction in R_0_ (**D**). As a result, maintaining infection at low non-increasing level within the control area requires continued follow up.

The invasion of ecosystems by non-native plant pathogens and insects [1], [2], [3], [4] poses a growing threat to ecosystem function and conservation as global trade, travel and environmental change create opportunities for introduction and establishment of exotic organisms [5], [6], [7], [8]. Cryptic infection (i.e. asymptomatic or undetectable for a period of time) and long-distance dispersal (i.e. with a fat-tailed probability distribution) of transmissible pathogens and pests present two serious impediments to the effective control of these organisms: the epidemics only become apparent once symptoms develop, by which time the outbreak will have grown, and new, sometimes distant foci may have been established through long-distance dispersal [9]. When the invading agents are unknown, they are likely to spread unnoticed and unchecked for even longer if their identification is difficult and their transmission poorly understood. The combination of delayed detection of cases with long-distance dispersal has the potential to sustain invasive spread even under modestly favourable conditions for the invading organism. In fact, management strategies that are restricted to the treatment of symptomatic hosts are likely to fail without offering much return for the resources deployed [10], [11], [12]. The challenge in devising epidemiologically- and economically-viable management strategies lies instead in matching the temporal and spatial scales of control with often poorly understood temporal and spatial scales of epidemic spread. Adopting such a scale-matching approach at a landscape level requires estimation of the actual spatial extent of the epidemic, including the location and speed of its expanding front. The degree of matching that can be achieved is determined by governing principles, regarding the location and nature of management actions, illustrated schematically in Fig. 1.

The problem of cryptic infection is not restricted to natural communities. For example, cryptic infection has frustrated efforts to eradicate the AIDS pandemic [13] and regional endemics of malaria [14]. However, in natural communities there are specific difficulties in matching scales of control with intrinsic epidemic scales. These difficulties include accessing sites to detect and control new infections, identifying the range of host species of a pathogen, and surveying the spatial distribution of hosts in a heterogeneous landscape. Within this context of uncertainty on multiple scales, the use of computational models for linking epidemiological, landscape and weather dynamics over large regions [15], [16], [17], [18] can assist in assessing the effectiveness of disease management strategies.

The use of models to inform disease management across landscapes can be divided into a number of interlinked stages: (i) construction of robust models that capture enough biological features to exhibit realistic dynamics; (ii) estimation of parameters such as dispersal distances and rates of spread from incomplete observations of infection; (iii) use of the model to predict the current extent of symptomatic and asymptomatic infection in order to assess the current status of damage and risk; (iv) given the estimated current status, use of the model to predict future pathogen spread under different management scenarios. Unavoidably, these steps are taken under uncertainties about host distribution and density, abiotic forcing such as weather, and responses of pathogen and host life-cycles to treatments.

In this paper, we study the efficacy of management strategies for controlling epidemics of forest pathogens with cryptic infection and long-distance dispersal, focusing on the emerging water mould *Phytophthora ramorum*, the cause of sudden oak death [19]. The current epidemics of sudden oak death, particularly in California, USA, are similar in extent and severity to historical outbreaks of white pine blister rust [20], chestnut blight [21], [22], [23], and Dutch elm disease [24], [25]. Limited understanding of pathogen transmission and biology, lack of multiple-scale epidemiological insight, and failure to recognize cryptic pathogen spread, contributed to the failure of attempts to manage these historical outbreaks [22].

We use computationally-intensive approaches for modelling and parameterizing spatio-temporal stochastic population dynamics in order to examine scenarios for the control of emerging epidemics in natural forest landscapes. We consider how to design efficient control strategies that account for uncertainties associated with cryptic infection and long-distance dispersal in heterogeneous host landscapes. Specifically, we focus on the control and management of sudden oak death in redwood-tanoak and Douglas-fir-tanoak forests of northern California. Our purpose is twofold. First, to illustrate a case study where availability of epidemiological and landscape data - typical of datasets that are or can be collected in natural ecosystems - allows modelling of pathogen spread and control, and offers advisory messages on current and future invasive organisms that are difficult to control. Our second purpose is to provide practical guidance for planning of control and prevention of further spread of *P. ramorum*, both in California and in temperate and coastal areas of Europe and the eastern USA where outbreaks have occurred in nurseries but spread in the wild has apparently been limited [26], [27], [28], [29]. Recent rapid spread in larch plantations in Britain and Ireland has been causing great concern and offers a cautionary example of the unpredictable impacts of this pathogen in new environments [30].

**Figure 2:**
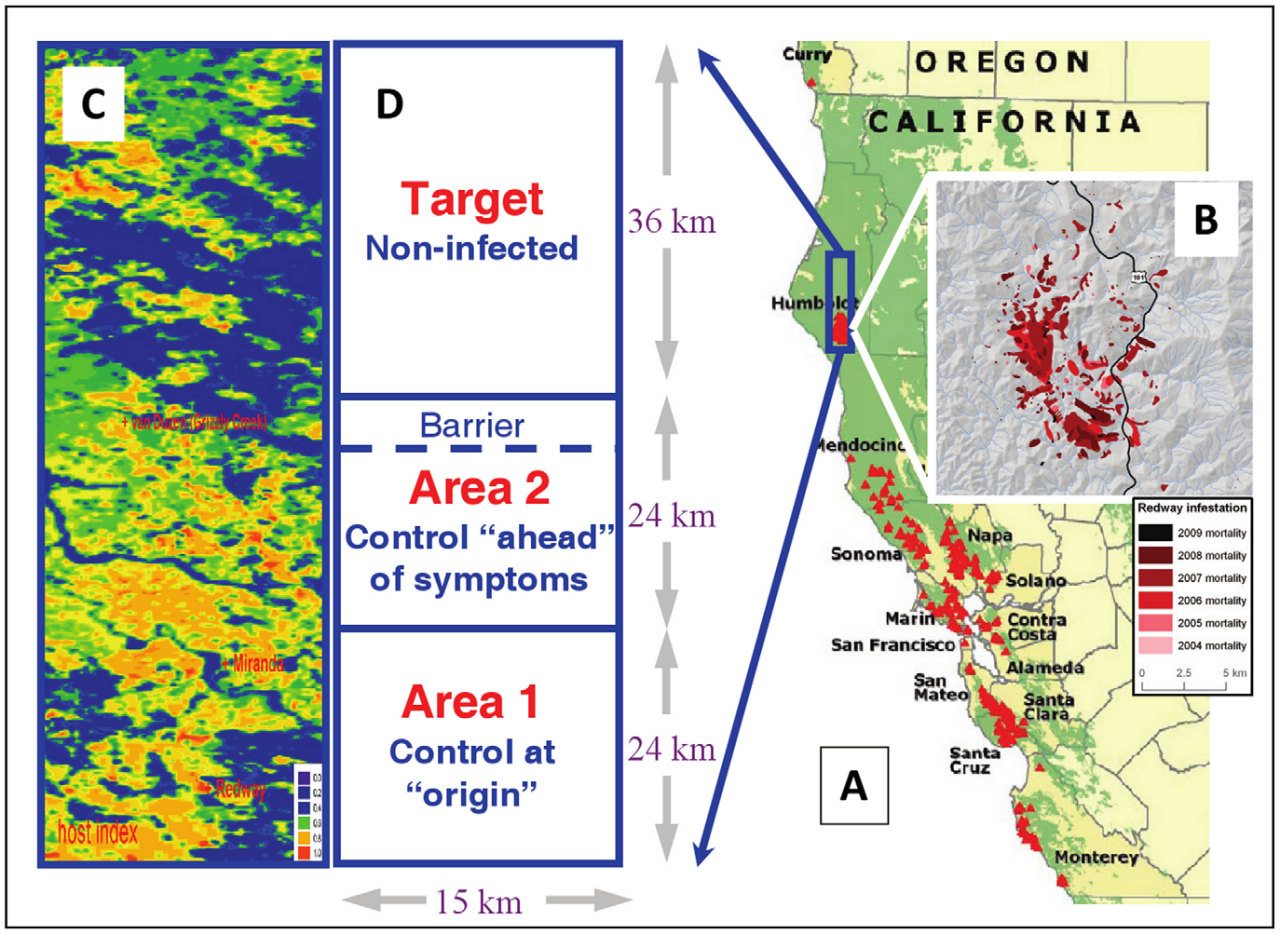
Study area, host, and mortality distribution in Humboldt County CA, USA. **A**) Humboldt county is shown in reference to sudden oak death distribution in costal California in 2008. **B**) Distribution of overstory tree mortality between 2004 and 2009 determined from annual aerial surveys; these data were used to determine dispersal and other epidemiological parameters of the causative agent *Phytophthora ramorum*. **C**) Host index within the 15-by-84 km study area. Scale: <0.01 (purple), 0.4 (blue), 0.6 (green), 0.8 (yellow), 1.0 (red); darker areas are dominated by non-sporulating or non-susceptible hosts while yellow and red areas are primarily dominated by tanoak (*Lithocarpus densiflorus*). **D**) Disease control areas and objectives, and approximate location of the proposed barrier treatments.

*Phytophthora ramorum* has been expanding its range in coastal California since the mid-1990s, killing millions of trees, including oak (*Quercus* spp.) and tanoak (*Lithocarpus densiflorus*, recently attributed to a new genus as *Notholithocarpus densiflorus*) [27], [31], [32]. This epidemic has caused damage to public and private property, economic impact on nursery, gardening and logging industries, and increased the cost of implementing regulatory activities [33], [34]. Many are also worried that large-scale tree mortality will have profound long-term environmental consequences, by changing the structure of plant and microbial communities, altering landscape ecological structure and function, and increasing forest-fire hazards [3], [27], [31], [35], [36], [37], [38]. *Phytophthora ramorum* is known to infect over one hundred species of forest shrubs and trees [27]. On oak and tanoak trees, *P. ramorum* causes bleeding bole cankers that can lead to relatively rapid mortality; therefore the disease name of sudden oak death. Other hosts such as California bay laurel (*Umbellularia californica*) suffer mild leaf-blight or twig-dieback symptoms and are major sources of inoculum for infection of oaks and tanoaks [39]. There is no evidence for sporulation of this pathogen from true oak species (*Quercus* sp) in Californian ecosystems [39]. Transmission of inoculum is thought to occur both locally and over long distance via rain splash, stream and river currents, wind and mist, and human-mediated transport [27], [28], [40], [41], [42]. The earliest symptoms of *P. ramorum*–invasion of a site are often small lesions on the foliar hosts, with minor effects on tree health and similar to lesions caused by native pathogens. Hence, confirmation of invasion by *P. ramorum* requires extensive on-the-ground sampling and laboratory isolation of the pathogen, which makes early diagnosis over large areas impractical. The alternative method of aerial surveying (Fig. 2) can only detect host mortality, which may be preceded by pathogen establishment by several years. These limitations in surveying are a primary cause for cryptic infections of this pathogen.

Control of *P. ramorum* poses significant epidemiological challenges: in addition to its cryptic and long-distance spread, the long infectious period and generalist nature of the pathogen aid its transmission across heterogeneous landscapes. Moreover, current measures for controlling *P. ramorum* at the landscape scale consist mostly of host removal, as no effective chemical treatment or biological control exists [27], [43], [44]. These measures are restricted by economic cost, logistics, and limited options for coordination with private landowners [27], ; they are also complicated by the disparate epidemiological, commercial, and amenity importance of the different hosts. In California, control of *P. ramorum* has been largely limited to state-wide quarantine [46], [47] and small-scale treatments [48] as the epidemic has grown rapidly on multiple fronts. Subsequently, desirable epidemic control via coordinated scaled-up treatment and prevention has been hampered by uncertainty about how to act effectively with limited resources, allowing the scale of the problem to continue to aggravate.

In the late 1990s, the *P. ramorum* epidemic took a geographic leap from the main focus around the San Francisco Bay Area, probably through human-mediated pathogen transport [40], to establish two disjunct outbreaks in northern California and southern Oregon (Fig. 2) [49].The Curry County, Oregon outbreak was first reported in 2001 [41]. Since the early stages of its detection, this outbreak has been kept under intensive control through extensive monitoring and removal of both infected host material and surrounding hosts as a buffer [41], . These aggressive treatments have contained, but not eradicated, *P. ramorum*, which has continued to spread within a relatively small geographic region, probably due to cryptic infection that makes early detection difficult [41], [48]. The pathogen has now been detected in scattered clusters in Curry County that add up to an area of about 80 ha [44]. However, the eradication attempts have prevented local intensification of the disease and minimized damage to the forest [44]. The other isolated outbreak, in Redway, Humboldt County, California was reported in 2002 [45]. Unlike the Oregon outbreak, eradication was not attempted and management has been of limited extent at the Humboldt County site; this outbreak has expanded in each subsequent year [51] (Fig. 2A–B).

We develop and parameterize a mathematical model for forecasting the spread of *P. ramorum* and assessing control options in the Humboldt focus, the northern forefront of the Californian epidemic where host and environmental conditions [49] favour spread over a large stretch of forest (∼200 km by 75 km) extending up to Curry County, Oregon (Fig. 2A). The model combines the key aspects of *P. ramorum* epidemiology with data on vegetation distribution [49] and weather variation, and shares features with a model we have developed for the spread of *P. ramorum* in California in the absence of disease control [32]. We use this model to explore the following control scenarios being considered by policy makers [27], [45]: removal of hosts, protective aerial spraying (an experimental technique [51], [52]), and construction of a host-free “barrier” [53]. Within each scenario, we adopt strategies with differing degrees of match between the scales (spatial and temporal) of the control and the spread of *P. ramorum.* The outcomes of all these measures are uncertain but critical, given the amount of resources at stake and the risk in case of failure or lack of implementation of control. A consequence of the non-intuitive dynamics arising from the intertwining of multiple scales of pathogen dispersal with cryptic and symptomatic infections (e.g., Fig. 1), is that some conclusions about *P. ramorum* control in California may not follow our expectations. For example: Should we remove hosts at or ahead of the outbreak focus? When would protective spraying be most effective? Which size of barrier would work? More generally, we demonstrate conditions for achieving effective control (either delay in spread or eradication) of plant pathogens or forest insects with cryptic and long-distance spread.

## Methods

### Epidemiological principles in landscape control of plant pathogens

Landscape epidemiology uses concepts from epidemiology and landscape ecology in order to understand natural and managed disease dynamics on a large scale, such as regional or continental scales [8], [15], [16], [17]. In applying strategies for the control of invading pathogens at the landscape level, we need to consider limiting principles that determine the maximum gain achievable and the minimum effort (and economic expenditure) required given the current state of the epidemic. The limits posed depend on the degrees of cryptic and long-distance spread of the specific pathogen within the host landscape, as well as on the goals of the intervention (Fig. 1). First, if the aim is *eradication* of a local outbreak, it is essential to match the spatial extent of the control area to that of the pathogen. For pathogens with cryptic infection and long-distance dispersal the extent of the epidemic is likely to be larger and increase faster than what estimates based on observed symptoms suggest. Second, if the aim of the intervention is *control in a particular area* of the outbreak, it is necessary to re-apply treatment to make up for partial coverage and partial effectiveness of each round and clearing reinvasion from non-treated infected areas. Finally, if the aim is to *protect a target* area (at-risk but not infested) we need to assess how extensively to treat in and around that area in relation to the distance to the advancing front. In all cases, it is essential to *estimate the full extent of infection*, including its moving front, and the *rate of pathogen spread* in order to control disease effectively [10], [12]. Other modelling studies have examined principles of pathogen invasion in heterogeneous landscapes [4], [17], [54], [55], but addressed animal diseases or pathogens that pose a different challenge to control than the combination of cryptic infection and long-distance aerial dispersal that we have considered.

### Humboldt outbreak case study

We focus on Humboldt County as a case study for three reasons. First, this outbreak of *P. ramorum* is geographically isolated [40] and has grown with minimal intervention, which offers an opportunity to estimate the natural spread of *P. ramorum* in the wild. Second, we estimate dispersal and transmission parameters from aerial survey data on pathogen spread (provided by the USDA Forest Service, Forest Health Protection) that are unique in California and elsewhere, in that they cover a whole, mostly non-managed outbreak area for several years (2004–2009) and avoid some of the incompleteness and biases that ground surveys inevitably encompass. This aerial survey dataset was verified in two ways. Field verification of aerial detections of the pathogen was done through ground monitoring by local scientists trained in the identification of *P. ramorum* disease followed when necessary by laboratory confirmation. Although it was not possible to apply this approach to every detected symptomatic tree, a high degree of confidence in the surveys is conferred by the following factors: verification was done after every annual survey; priority was given to edges and outliers in the spatial pattern of detection; patchiness in tree mortality provides a strong signature of true detection at stand level. The aerial records were also checked by comparison with field observations from a permanent study-plot network and an early-detection watershed-level survey based on pathogen baiting in streams and rivers [38], [56]. In addition, we did a systematic validation of aerial detections against the presence of suitable hosts of *P. ramorum* using vegetation distribution databases such as CALVEG [57]. The third reason for using Humboldt County as a case study is that the isolation of the outbreak offers opportunities for control and requires management decisions that are specific to this outbreak. *Phytophthora ramorum* infections were first reported in Humboldt County in 2002 around the town of Redway [45]. The affected area in Humboldt has since then grown at an increasing rate with the mortality of tanoak and oak trees scattered over thousands of hectares (Fig. 2B, and Fig. S4 in Text S1). Tanoak mortality peaked in 2007 and has slowed since, likely due to low spring-rainfall from 2007 until 2009 (Fig. S1B in Text S1). The disease has spread predominantly northward of the initial focus near Redway, probably due to prevailing winds. To date, only moderate, localized control measures have been applied in Humboldt with evidence that they might have had an impact at (but possibly not beyond) the scale of the treated individuals and plots [45], [51]. The geographic isolation of this focus from the wider epidemic initially raised hopes of eradication [45], but the current size of the focus suggests that amelioration and containment are more realistic goals. No direct measures of the area with cryptic infection in Humboldt (which is wider than the area with symptoms) exist, because of the large spatial extent of the region that would need ground surveying for the presence of the pathogen, the limited resources to do so, and the spatial heterogeneity in landownership and in landowner cooperation with monitoring efforts.

### Control scenarios explored

Options for controlling *P. ramorum* are currently limited to removal of inoculum (i.e., culling and burning of diseased hosts), removal of hosts (i.e. pre-emptive host culling with herbicide or cutting), and chemical protection; but no curative chemical treatment or biological control exists [27], [43]. In each of these approaches there is difficulty in field identification of infected hosts, treatment costs are high, treatment permits are slow to obtain, and the logistics of working in areas with many small landowners complicate the implementation of treatment. We explore the following control strategies initiated in 2010 and implemented in differing spatial areas (Fig. 2D). **1)** ***Removal at the origin***, in an area containing the focus (*Area 1*, Fig. 2D) about once per year; this strategy includes follow-up monitoring (more frequently in cells with more abundant hosts), partially-effective detection of symptoms, and removal of inoculum and hosts in symptomatic (and adjacent) stands using host removal, herbicide treatment and pile burning [27], [51]. **2)** ***Removal ahead of the origin***, in an area north of the focus (*Area 2*, Fig. 2D); is otherwise identical to ‘removal at the origin’. **3)** ***Mixed strategy***: Aerial spraying with Agri-Fos® (a phosphate compound) [43] on a large scale [52] to provide temporary partially-effective protection of hosts (e.g. tanoak) and prevent northern spread (to the *Target*); here we combine inoculum ‘removal at the origin’ (*Area 1*) and, with the same frequency, spraying ‘ahead of symptoms’ in areas with lower human-population density (*Area 2*). While some aerial spraying experiments are ongoing in Oregon, the long-term efficacy and practicality of these treatments has not yet been established. In California, it is likely there would be limited willingness of landowners to approve aerial spraying, which would impede large-scale host protection treatments in Humboldt. Therefore, we explore this control scenario as a hypothetical investigation of the impact of altering forest susceptibility at landscape level. **4)** A ***host-free ‘barrier*** **’**, an approach initially proposed for the vicinity of Redway when the disease focus was smaller [45], but here located further north just south of Grizzly Creek, a tributary of the Van Duzen River watershed, to prevent northern spread (to the *Target*, Fig. 2D); a similar barrier has been under construction a few kilometres north of the location we consider in the model [53]. For all scenarios, we concentrate on a region containing the initial focus near the south edge and extending ∼85 Km north (Fig. 2C–D), the predominant direction of spread. Control is implemented and ‘northern invasion’ defined according to a breakdown of this region into *Area 1* (comprising the focus), *Area 2* (north of the focus and predicted to contain less or no infection at the start of control), and the *Target* area (predicted not to be infected at the start of control and to be protected from invasion). We study different spatial scales of control, i.e., the size of Area 1 (equal to that of Area 2) in relation to the spatial extent of cryptic infection (set by the location of the epidemic front). The ‘barrier’, located at the north edge of Area 2, extends from east to west, is either 5 km or 10 km wide north to south, and is managed in order to remain host free. We run the control scenarios from 2010 to 2017 and, with an earlier start date, from 2005 to 2017. Host removal and spraying are implemented roughly synchronously across the control area to optimize impact, and followed up to account for incomplete detection and partial coverage and effectiveness of treatments.

### Model description and estimation

We developed a probabilistic, spatially-explicit metapopulation model for the transmission dynamics of *P. ramorum* in a landscape of mixed-host stands represented by square cells (250 m by 250 m). Each cell has a susceptibility and infectivity that were evaluated based on its composition and density of host species, estimated using the CALVEG database of plant community distributions [57] implemented in a geographic information system (GIS) [49]. At each time, a cell can be in one of four states: Susceptible; Infected and asymptomatic (cryptic); Infected and symptomatic (detectable); or Removed (where treatments are applied). Removed cells can be re-colonized via host re-sprouting or host re-invasion. Infected cells can transmit inoculum to susceptible cells according to a dispersal kernel (a probability function of relative distance) and have an average infectious period of 10 years. Several studies suggest that the infectious period of *P. ramorum* is limited (albeit long) and varies among species and environmental conditions [39], [58], [59]. In bay laurel, leaf shedding rates increase in the presence of foliar infection with a greater increase in dry than in cool and humid conditions [58]. These observations suggest a mechanism by which non-lethal hosts can recover from infection and limit their infectious period. In tanoak twigs and stems, no mechanism of recovery from *P. ramorum* infection is documented, but systemic infection is lethal in this host and observations suggest that no sporulation occurs on dead tanoak tissue [59]. Therefore, we assume a finite infectious period that is longer than the time since annual surveys of *P. ramorum* were initiated in California [38]. The effects of variable spring-rainfall and temperature on pathogen transmission through sporulation and infection [39], [58], [59] are accounted for in the estimation of the model parameters and in the predictions. The model was parameterized using aerial surveys of tanoak mortality in the Redway area between 2004 and 2009. We applied Bayesian Markov chain Monte Carlo, data-augmented inference [60] to estimate the time and location of the index case (year 2001, 2–3 km south of Redway), the rate and ‘spatial scale’ of transmission, and the rate of disease-induced tanoak mortality. The estimated average time between tanoak infection and mortality is about 2.5 years ([2.3, 2.9] 95% credible interval). In addition, we used this inference procedure to choose among candidate dispersal-kernel functions, which potentially can greatly influence the predictions of pathogen spread and of the impact of management strategies, as hypothesized at the beginning of the paper. We found that *P. ramorum* can disperse over large distances with a long tail of low probability: a power-law function fitted the data significantly better statistically than a negative-exponential function. We also contrasted the goodness-of-fit of the models based on each of the dispersal kernels through a visual comparison of predicted and actual progress of disease in space and in time (Fig. S3 and S4 in Text S1). We note that it was not possible to cross-validate the model against independent representative data because no such data were available. For some of the control scenarios explored, we calculated a local basic reproduction number (R_0_) to assess the impact of treatment in the area where control is applied (Fig. 1). The local R_0_ is determined by the estimated dispersal kernel and transmission rate of the pathogen and by the post-treatment host-landscape. See Text S1 for further detail on model assumptions and formulation, estimation methods, and predictions.

### Range of scenarios for pathogen spread and effectiveness of treatments

In order to probe the generality of the model outcomes, we considered three scenarios representing a likely range of ability or risk of the pathogen to spread in the host landscape: “high”, “medium” and “low” pathogen-spread scenarios. We defined these scenarios using the inferred uncertainty about the three estimated parameters characterizing pathogen transmission and the period of cryptic infection. We may think of this ability or risk to spread as a pathogen trait encompassing the joint effect of several traits and factors. The medium-spread scenario corresponds to the median of the posterior distribution of the estimated parameters; this is the case considered in all results presented, unless stated. The high (low) spread scenario corresponds to a combination of parameter values leading to greater (lower) potential of the pathogen to spread and lower (higher) efficacy of practitioners to detect infection; we chose these values based on 95% credible regions of the parameters. We use these pathosystem scenarios to study how the impact of control strategies depends on our estimates of epidemiological parameters (Text S1), and to assess whether the predictions in the “medium-spread” scenario are representative, or could change under uncertainty about parameters or conditions presented by other host-pathogen systems.

## Results

### Predicted natural spread

**Figure 3:**
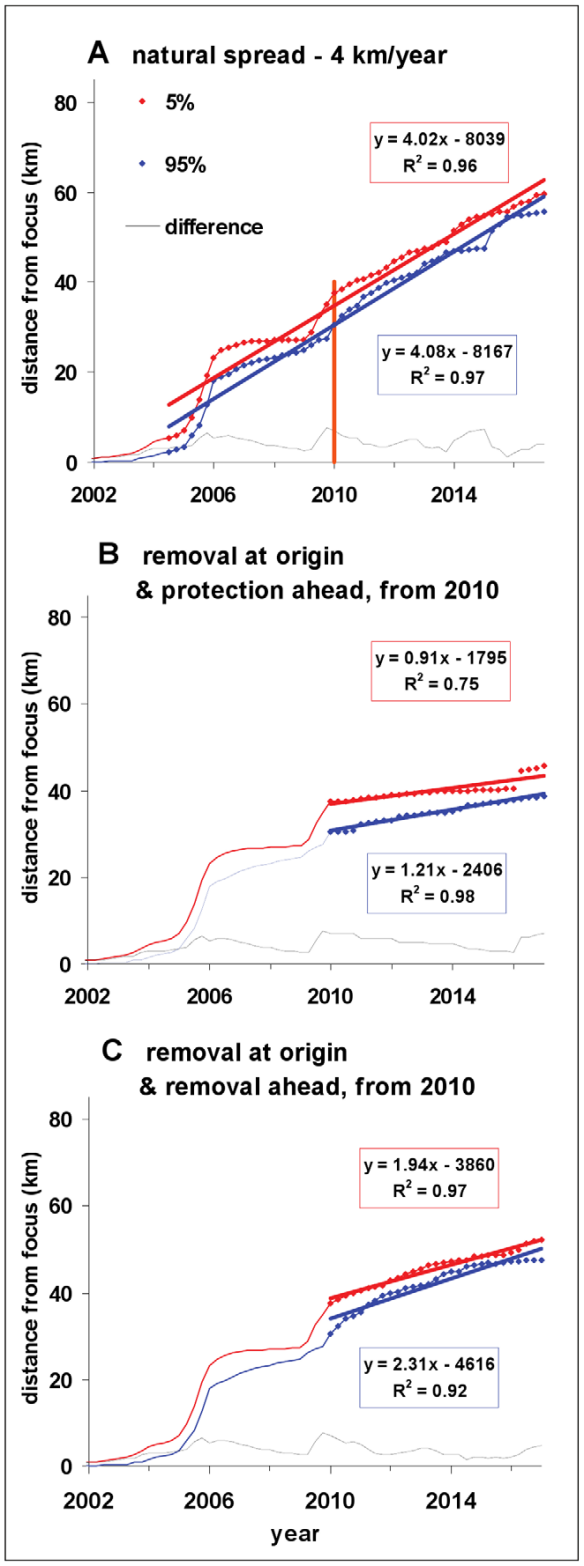
Speed and location of the cryptic epidemic front in Humboldt County up to 2017. **A**) We predict that the front moves north of the focus with average speed ∼4 km/year and in 2010 is located 28 to 35 km north of Redway (i.e., 31 to 38 km north of the estimated centre of the focus; see orange vertical line). If control were initiated in 2010 the front would slow down to: **B**) ∼1 km/year, with removal “at the origin” and host protection “ahead of the origin”, although in 2016 the front would jump over the protected area and speed up (Fig. 4E); and **C**) ∼2 km/year, with removal “at” and “ahead of the origin” (Fig. 5D). The annual weather pattern in each year after 2010 equals the average during 2000–2009. The red and blue curves show the locations where the probability of pathogen invasion is 5% and 95%, respectively, which we use to define the moving front of the epidemic. The speed of the moving front is bounded by the slopes of the straight lines that approximate the iso-probability curves. The position of the front at given time is bounded by these curves; the distance between them (black line) provides a measure of uncertainty associated with chance variation in the spread of infection. When there is control, the lines are fitted to the post-control period to avoid influence by past conditions, while for natural spread a broader period is allowed including past and future.

First, we forecast the current size of the epidemic (including cryptic and symptomatic infection) and the current and future speed of its moving front under natural conditions, i.e., without management actions. This step is essential as observations of disease symptoms do not reveal the full extent of the infection focus. We define the epidemic front as the stretch of landscape where the probability of invasion changes from 95% to 5% as the distance from the focus increases. Assuming that the weather pattern in each year after 2010 equals the average of annual patterns during 2000–2009, we predict that the epidemic front will advance northward at a speed of about 4 km/year (Fig. 3A). The speed of the infection front is driven by weather and landscape conditions that affect the pathogen: it was slower before 2004 when the focus contained few unit cells, and during 2007 and 2008 when weather (Fig. S1B in Text S1) and local landscape conditions were less favourable for infection, but faster in 2005 and 2009 when these conditions were favourable; the predicted slow down in 2013–14 is due to naturally-lower landscape-level contiguity of hosts in the northern part of the study region (Area 2, Fig. 2). We forecast that in 2010 the front of the epidemic is situated 28 to 35 km north of Redway, between Miranda and the Van Duzen River in Humboldt County. These predictions do not account for heterogeneity in topography, which could affect spread. In addition, the predictions are likely to be sensitive to future change in annual weather and climate [39], [59], e.g., caused by changes in the strength and duration of future El Niño/La Niña oscillation cycles, as suggested by the effects of past weather variability on model output (Fig. 3A). It is possible, therefore, that future surveys and weather would yield different estimates of epidemic front dynamics. For example, our preliminary estimates based on data up to 2007 [61] yielded a faster advance of the front. Nevertheless, the predictions in Fig. 3A provide the best estimates available on current evidence.

### Predicted impact of control strategies

**Figure 4:**
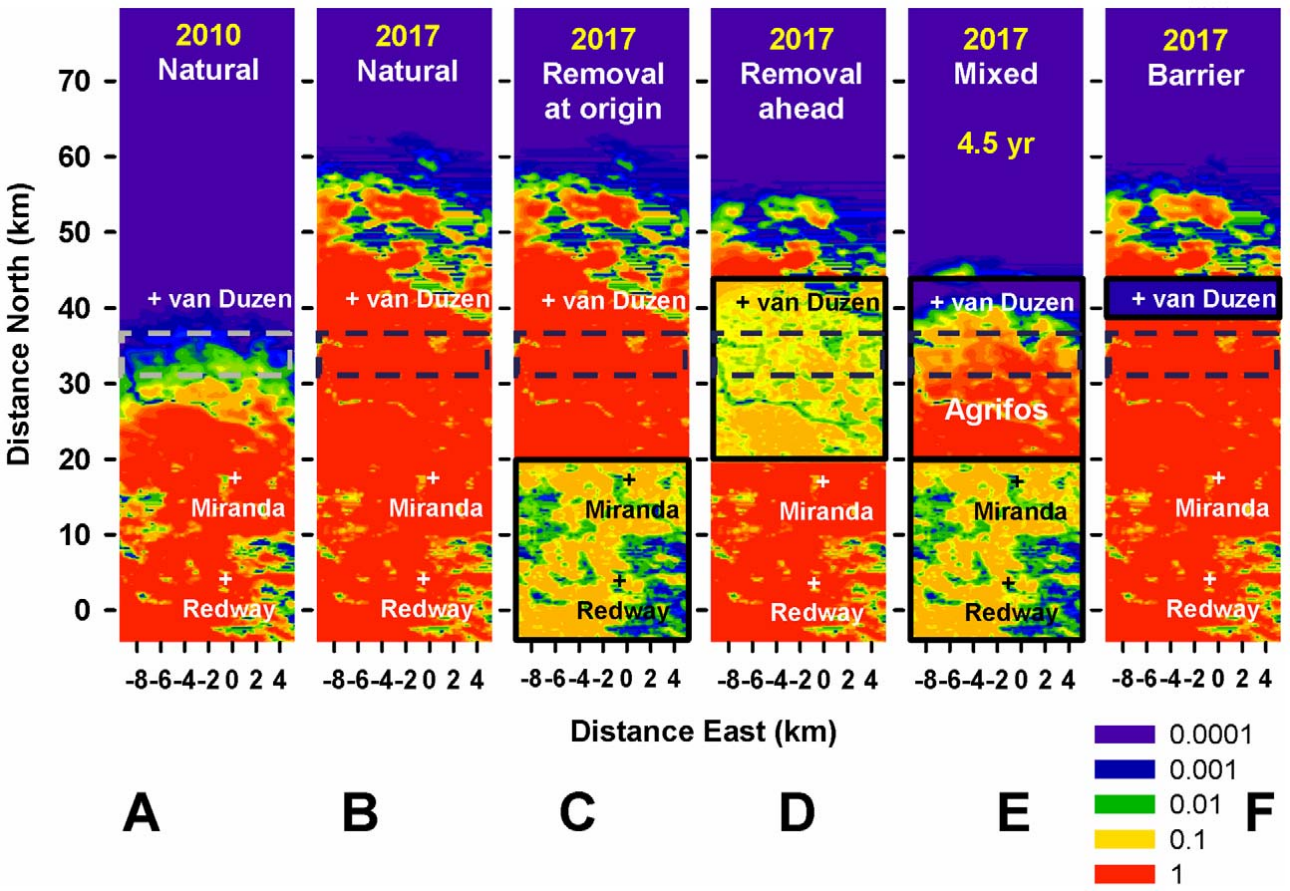
Alternative treatments initiated in 2010 in areas smaller than the cryptic epidemic. Risk maps showing probability of infection (cryptic and symptomatic) on logarithmic scale (red∼1, yellow∼0.1, green∼0.01, blue∼0.001, violet≤0.0001). In 2010 the epidemic front is 31–38 km from the origin (broken lines, c.f. Fig. 3). **A–B**) 2010 and 2017: natural spread. **C–F**) 2017: controlled spread – all treatments fail to contain the front and protect the *Target* area from invasion; the delay in invasion is indicated (top) where ≥1year. **C**) Removal at the origin (Area 1, thick black line, c.f. Fig. 2D) – the front is not delayed significantly (c.f. Fig. 1C); local inoculum is kept at a low level but is not eliminated due to the 2–3 year delay in detecting cryptic infection and removing inoculum, host re-colonization after removal, and re-infection from non-controlled-areas (c.f. Fig. 1D). **D**) Removal ahead of the origin (Area 2) – the front is also not delayed significantly. **E**) Mixed strategy: host protection (Agri-Fos®) ahead of the origin and removal at the origin – the protection initially extends beyond the epidemic front and delays it (speed∼1 km/year, Fig. 3B), but as protection is partial and wanes, this “barrier” thins (c.f. A) and is overcome by long-distance dispersal (c.f. Fig. 1E). **F**) “Host-free barrier” 5 km thick, 35 km from Redway, is overcome by long-distance dispersal (c.f. Fig. 1E).

Sustained removal of inoculum on a smaller scale than the size of the epidemic focus at the start of control – either “at” or “ahead of” the origin (Area 1 or Area 2, <16 km or <40 km north of Redway) – is effective locally but fails to contain or delay invasion of the Target area (Fig. 4C–D) due to spread from undetected cryptic infection. Despite the local basic reproduction number *R_0_* dropping from >10 to <1 in either control area, elimination is thwarted by re-infection from non-controlled areas (Fig. S5 in Text S1). There is a marginal advantage in treating ahead rather than at the origin, because only part of Area 2 is infected in 2010, which allows for a delay in spread of infection within and beyond it. Supplementing the removal at the origin with host protection (Agri-Fos® spraying) that stretches a few kilometres beyond the epidemic front (Fig. 4E) slows down the epidemic front from 4 to ∼1 km/year but fails to contain it. Indeed, the front re-gains speed in 2016 (Fig. 3B) as protection wanes (and some susceptible forest is infected before the next spraying round) and mounting inoculum disperses over this thinning “barrier”. Fig. 4D–E, 4C and 4F provide examples in support of the principles stated in Fig. 1C, 1D and 1E, respectively, i.e., invasion ahead of the treatment area, re-invasion of the treated area, and spread over barriers.

**Figure 5 pcbi-1002328-g005:**
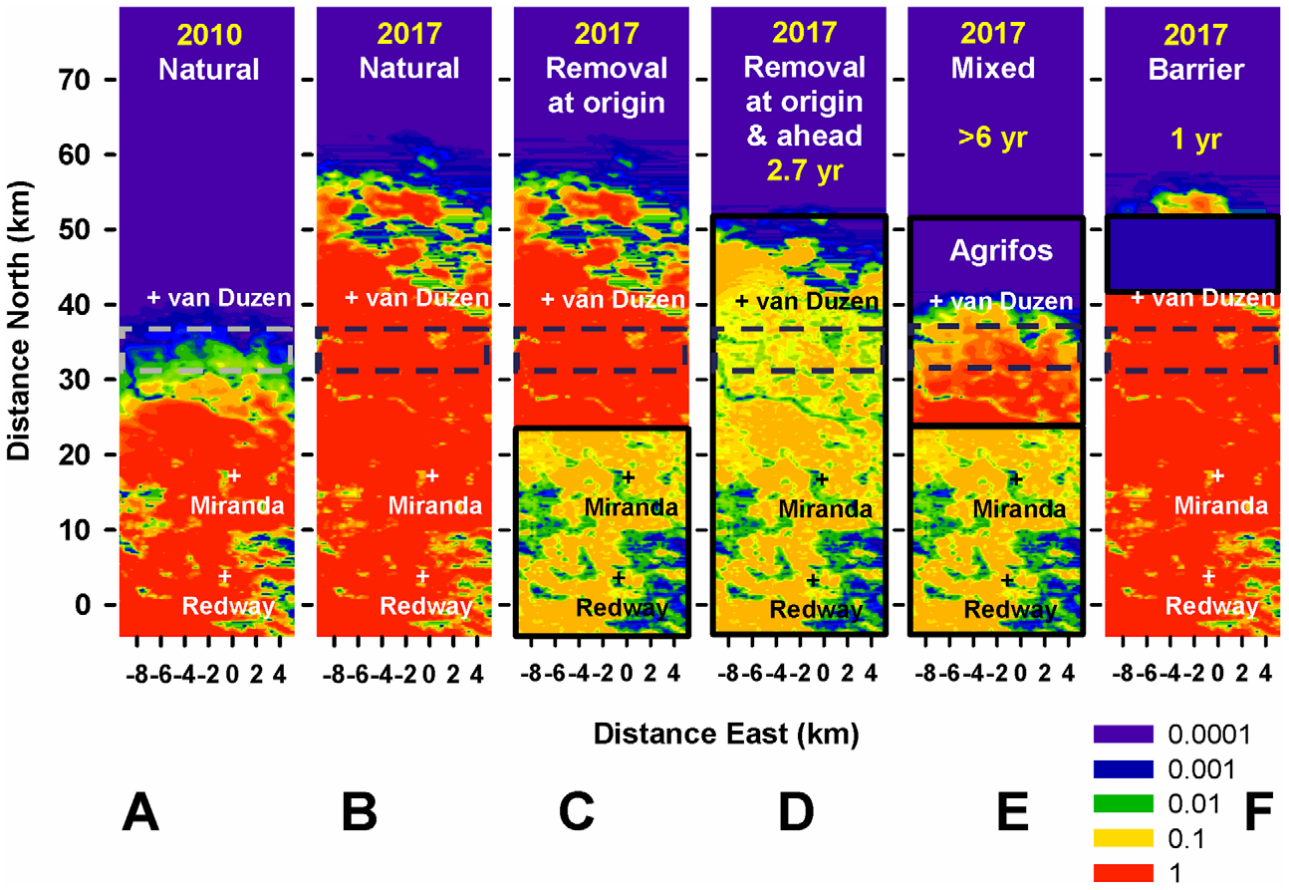
Alternative treatments initiated in 2010 in areas larger than the cryptic epidemic. Risk maps as in Fig. 4, but control areas are 4 km bigger (C–E) and the “barrier” (F) is twice as thick and 3 km further north. **A–B**) 2010 and 2017: natural spread. **C–F**) 2017: wider control has a mixed outcome. **C**) Removal at the origin – still covers an area smaller than the initial cryptic epidemic and has limited impact, as in Fig. 4C. **D**) Removal at and ahead of the origin – covers and extends beyond the infected area and delays epidemic progress significantly (speed ∼2 km/year, Fig. 3C); cryptic infection is visible (top edge of removal area) where it is more intense because it is too recent to be detectable and removed. **E**) Mixed strategy – covers and extends beyond the infected area and delays epidemic progress significantly (speed ∼1 km/year, Fig. 3B); protection is more effective (and less host-damaging) than with extended removal (D), although allowing for higher inoculum levels, and contains spread to the Target area, unlike the smaller-scale control (Fig. 4E). As in Fig. 4, inoculum in C–E cannot be brought down further due to the delay in detecting cryptic infection and in subsequent removal (c.f. Fig. 1D). **F**) Larger, 10 km thick “host-free barrier”, 38 km from Redway – is overcome through build-up of inoculum and long-distance dispersal, but delays invasion of the Target area by ∼1 year (c.f. Fig. 1E).

**Figure 6:**
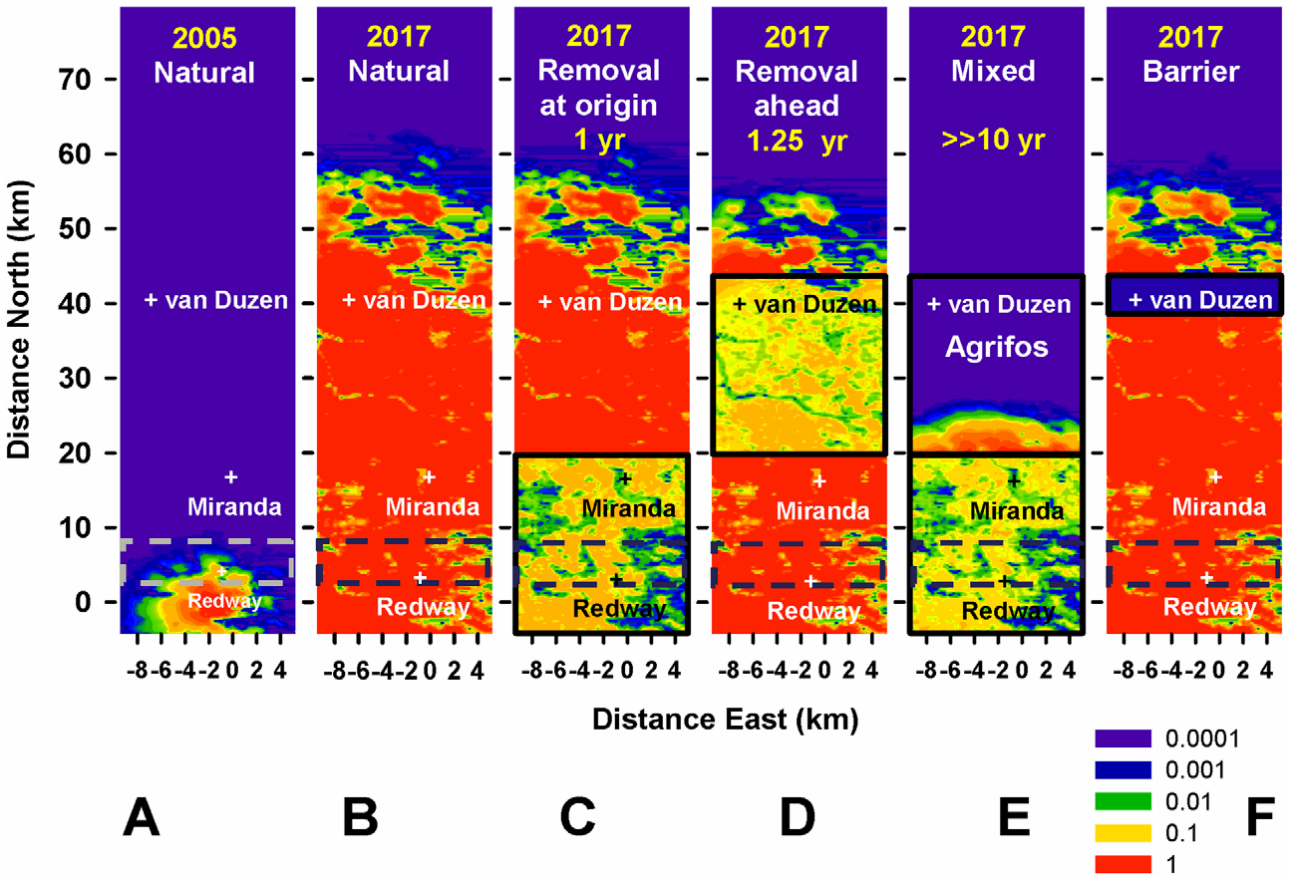
Alternative treatments initiated in 2005 in areas larger than the cryptic epidemic. Risk maps as in Fig. 4, but control starts 5 years earlier when the cryptic epidemic is much smaller. **A–B**) 2005 and 2017: natural spread. **C**)**–F**) 2017: earlier control has the greatest impact. **C**) Removal at the origin – the front is delayed more and local inoculum is reduced more than with control initiated later (Fig. 4C), but it is still not eliminated due to cryptic infection and re-infection (c.f. Fig. 1D). Removal initially covers a larger area than the cryptic epidemic but once the front passes the edge of this area it spreads nearly as fast as without control, limiting the overall delay to ∼1 year. **D**) Removal ahead of the origin – as in C, the front is delayed more and local inoculum reduced more than with control initiated later (Fig. 4C) but it is still not eliminated. Removal reduces the mass of inoculum nearer the Target area and delays the front slightly more than removal at the origin (C). Cryptic infection is visible in the top edge of the removal area. **E**) Mixed strategy – covers a larger area than the cryptic epidemic and protection is applied before there is any infection in the protected area delaying the front much more than control initiated in 2010 (Fig. 4E). **F**) “Host-free barrier” – identical effect to Fig. 4F because the barrier is well ahead of the front both in 2005 and in 2010 (c.f. Fig. 1E).

Sustained removal (either alone or with host protection) on a scale larger than the size of the epidemic focus at the start of control – either by increasing the size of the control area (Fig. 5) or through early monitoring and treatment (Fig. 6) – controls infection locally and delays invasion of the Target area significantly (≥1 year). First, removal starting in 2010 in an expanded area stretching well beyond the epidemic front (∼15 km) reduces the overall level of inoculum and delays invasion of the Target for ∼3 years (Fig. 5D). If removal had started in 2005 in the original, smaller area it would have delayed invasion of the Target by ∼1 year (Fig. 6C). As above (Fig. 4) there is a marginal advantage in treating ahead of (Fig. 6D) rather than at the origin (Fig. 6C) because inoculum is reduced nearer the front. The likely reason why removal yields only modest delays, even when expanded in space or time, is the delay in detection of infection within the control area due to the cryptic-infection period (>2 years). Removal treatments alone do not cause a sufficient drop in inoculum to slow down the front significantly, although the drop is greater with control starting in 2005 (Fig. 4 and 6C–D).

Second, large-scale protection/spraying ahead of the origin, together with removal at the origin, starting in 2010 in an expanded area slows down the front speed to ∼0.5 km/year and prevents invasion of the Target for >6 years (Fig. 5E). If this mixed strategy had started in 2005 in the original, smaller focus the entire host-protected area would have been infection-free initially. While the front speed would have decreased to about the same level as with an intervention starting in 2010 (∼0.5 km/year), the extent of host protection would have been maximized and invasion of the Target contained for a much longer period, well over 10 years (Fig. 6E).

A 5 km wide host-free “barrier”, just south of the Van Duzen River (Fig. 4F), is ineffective at containing spread because inoculum builds up behind the barrier and occasional long-distance dispersal eventually succeeds in establishing new infection foci north of the host-free zone. A 10 km (rather than 5 km) wide barrier (located 3 km further north) (Fig. 5F), also fails to contain spread overall but is successful in delaying spread for about one year.

Overall, the control strategies involving removal or chemical protection of hosts slow down rather than interrupt spread due to the partial coverage and efficacy, and the limited temporal duration of the treatments, e.g., not all hosts in a control area are treated and the effect on those that are treated is partial and temporary. Regarding where to apply curative treatment, the best location for removal depends on whether the goal is reduction of existing infection or containment of its spread. Both preventive measures, host chemical protection and the “host-free” barrier, need to be applied ahead of the front and the bigger the protected area or the wider the barrier the greater the impact. Removal is the only curative treatment for *P. ramorum* in the ecosystems of the western USA, and as such is the only treatment capable of reducing inoculum where it is already present (e.g. Fig. S5 in Text S1).

### Range of scenarios for pathogen spread and effectiveness of treatments

The predicted epidemic growth over time, in the absence of interventions, over the range of pathogen-spread scenarios (high, medium and low spread) distributes approximately evenly about the medium-spread scenario and the survey data (Fig. S6A in Text S1). This evenness suggests these scenarios are representative of a likely range of epidemic potential associated with the inferred uncertainty in the estimated parameters. The differing ability of the pathogen to spread in each of these scenarios influences the relative impacts of control strategies in an expected way. As the spread potential of the pathogen increases, the invasion of the non-infected area (Area 3, Fig. 1) is delayed increasingly more (Fig. S6B in Text S1). Moreover, the ranking of the different control strategies according to their impact is preserved across the range of potential pathogen-spread scenarios (Fig. S6C–E in Text S1). We conclude that results comparing the effectiveness of control strategies in the medium-spread scenario (Fig. 3, 4 and 5) are qualitatively robust and representative of the viability of these strategies over more general conditions, including potentially other host-pathogen systems. Note that the measures of impact of the two removal strategies, “at” and “ahead of the origin”, crossover in the course of time because the outcomes of the strategies are case sensitive, as already stated. The mixed strategy is sustainable (i.e., the infection level remains stable in the long term) in the medium- and low-spread scenarios (Fig. S6C in Text S1), while removal over an enlarged area (larger than the cryptic epidemic, Fig. 5) is sustainable in the low-spread scenario.

## Discussion

Faced with an invading plant pathogen, it is vital for the success of control to identify the pathogen's biological and epidemiological features early on by collecting adequate field and laboratory data. The invading pathogen may be the cause of an established outbreak, such as *P. ramorum* in California and more recently in Western Europe, or an emerging threat, such as the risk of *P. ramorum* establishing in other areas. If the pathogen has cryptic spread and/or long-distance dispersal it presents non-intuitive multiple-scale dynamics that make it difficult to anticipate the impact of landscape management strategies and require re-evaluation of conventional approaches to regulatory and control activities [10], [11], [12], [46], [47], [61]. For example, are expectations about the impact, timing, and location of treatments (which are likely to have logistical delays and sparse spatial coverage) justified? For *P. ramorum*, cryptic infection makes it very difficult to identify the actual extent of outbreaks; which, combined with the pathogen's long-distance dispersal and long infectious period, leads to much uncertainty about an effective strategy for eradication or at least for containment of this emerging pathogen [41], [48]. Historic epidemics of single-host pathogens with similar ability to spread cryptically have frustrated management actions in Europe and North America and caused extensive changes in natural and urban forest landscapes [21], [23], [24], [25]. Currently, emerging generalist pathogens such as *P. ramorum* and *P. cinnamomi*, the cause of jarrah dieback [62], [63], endanger plant species in North America, Europe, Australia, and South Africa, prompting a global need to understand their dynamics and to identify effective management.

By exploring options for the control of *P. ramorum* in northern California, we have demonstrated general principles for effective landscape control (containment or eradication) of forest pathogens characterized by cryptic and long-distance dispersal: **1)** Continued monitoring of an at-risk target area is essential for early detection and prompt action. Our model shows that if treatment is not followed up its benefits will not be sustained (Fig. S5 in Text S1). **2)** *Curative* treatment (e.g., removal) or *preventive* treatment (e.g., chemical or pre-emptive culling), should, respectively, be applied rapidly on the scale of the whole infested area, including cryptic infections, or in a large-enough non-infested area that includes the host landscape at significant risk. Our model shows that that treatment in a limited area can be rapidly overcome by re-invasion through long-distance dispersal from non-treated areas. **3)** If the control area is smaller than the infected area and there is long-distance dispersal, removal can be more effective “ahead of the origin” or “at the origin” depending on multiple factors, such as the extent of invasion “ahead of the origin”, the heterogeneity of the landscape, the tail of the dispersal kernel, and the efficacy of treatment. The choice of where to target treatment is highly debated by practitioners, including forest managers; our model suggests that this choice has to be made on a case by case basis. **4)** The scale of the pre-control cryptically-infected area must be predicted using epidemiological data, and preferably also a parameterized model; this scale depends on the dispersal kernel and on how long the pathogen has been established. **5)** Treatments should be applied as synchronously as possible across the control area to maximize the impact of resources and minimize pathogen escape, and should have repeated rounds to fight re-lapse of infection due to partial effectiveness, partial coverage, and (if applicable) re-infection from outside the control area. Our results indicate that partial coverage and lack of coordination (Fig. 4–6), or delay in follow up of treatment (Fig. S5 in Text S1) can drastically and rapidly reduce the impact of management strategies, resulting in potentially very low cost-effectiveness of these actions. **6)** Early treatment, when the expanding focus is smaller, is more cost-effective in achieving local control and protecting non-infected areas. Our results show that the growth in the extent of the cryptic epidemic can be much faster than what the visible epidemic suggests (Fig. 4 and 6). **7)** If control is applied late when the epidemic focus has grown significantly large, the more feasible goals are local reduction of inoculum and containment (e.g., years of delay in spread, Fig. S6B in Text S1), both of which can ameliorate local damage, may involve re-forestation, and could allow time for development of more-effective control tools. **8)** Host-free “barriers” of plausible width can be ineffective at containing long-distance dispersal, unless there are additional buffers of spread (e.g., topographic features). However, wider barriers (∼10 km, in the current study) can delay the epidemic front. Barriers have been proposed for controlling animal diseases [55], but are less likely to be successful with aerially-dispersed plant diseases. The above principles extend, in essence, to the management of other forest pests, such as wood-boring insects, which have had increasing economic and ecological impact [64], [65].

Unreliability in parameter estimates can affect our confidence in the predicted viability of control and management strategies. We found the results from our specific study, however, to be qualitatively robust to the inferred uncertainty in the parameters. For a generic host-pathogen system, there is uncertainty in the predicted efficacy of control strategies that involve removal of inoculum or host protection due to uncertainty in the pathogen dispersal kernel and transmission rate (parameters α and β) and in the model components representing the effects of the heterogeneous landscape and variable weather, all of which determine the pathogen's potential to spread and the severity of outbreaks. There is also uncertainty in the predicted impact of host protection, which depends on the duration of cryptic infection (related to parameter r_C_), and in the predicted impact of removal of inoculum, which depends, in particular, on the rate of host re-invasion of treated stands. We expect the efficacy of a host-free barrier to depend chiefly on the tail of the dispersal kernel. The viability of a barrier would be affected also by directionality and extreme strength of winds, but these factors would most likely reduce the efficacy of the barrier even further, while topographic features could have the opposite effect (c.f. Results).

For forest pathogens, there are specific challenging steps in designing management strategies, such as acquiring host and pathogen landscape distribution data [40], [56], determining the effects of environmental conditions on inoculum production and establishment [39], [66], and developing techniques to estimate pathogen dispersal parameters and the extent of cryptic infection. However, forest diseases have some simplifying features relative to, for example, annual herbaceous-plant communities or contact structures ruled by individual movement and behaviour in animal and human populations: forest trees are long lived and do not move. These factors lead to comparatively slower changes in community-level inoculum production and host composition as hosts die [67] and to more straightforward short term forecasting. Yet, an important limitation in modelling forest diseases is the lower volume and greater biases in case recording compared with standard collection of clinical and veterinary data on human and livestock diseases. Similar issues apply to the predictive modelling of cryptically spreading forest pests.

In relation to the sudden oak death outbreak in Humboldt County in northern California, our results suggest that *P. ramorum* will continue to spread north relatively rapidly in the medium and long term in the absence of effective landscape-level interventions. Spread on such scale could cause great damage in northern California, and eventually foil management attempts in Oregon [41] through the import of inoculum from uncontrolled epidemic foci. If extensive interventions were implemented, removal of inoculum on a sufficiently-large scale and frequency could delay the northern spread of the pathogen by several years. If this measure were supplemented with effective host protection (a form of “vaccination”) applied repeatedly ahead of the epidemic front, it could contain the spread for even longer. Large-scale chemical protection against *P. ramorum* is only at the very early stages of efficacy evaluation [52] and there would be substantial social, legal, and economic obstacles to its application in California. However, we explored this hypothetical control scenario to illustrate the potential impacts of changes in the epidemiological characteristics and spatial arrangement of hosts on the spread of *P. ramorum*. Our study suggests that the removal of infected hosts could be much more effective in ecosystems where landscape-level host communities are (or have been made) less susceptible to infection or support lower rates of sporulation. While we have demonstrated the importance of the epidemiological characteristics of host communities by considering reductions in susceptibility and sporulation of hosts that result from hypothetical chemical treatments in northern California, the implications of our results extend to other locations at risk of *P. ramorum* emergence such as eastern USA forests and parts of Europe. In such locations host characteristics might differ and/or it could become feasible to apply a form of extensive protection treatment in the future.

While containment of the pathogen in southern Humboldt County is possible in theory, the estimated large size of the focus and potential long-distance dispersal of *P. ramorum* make the scale, nature of treatment, and coordination needed to do so a major challenge. Moreover, we find no evidence that a host-free “barrier” would contain the pathogen's dispersal for a significantly long time, at least under the assumption of similar topographic and weather conditions to those near Redway, the source of the epidemiological data used to parameterize the model. Nevertheless, although our results suggest that *full* containment is not likely, they also suggest that removal of infected hosts can reduce inoculum effectively within a control area and yield local benefits. This outcome is important for the implementation of policy on disease management and regulatory control, because removal of infected hosts is the only established means of treating infection by this pathogen. Moreover, applying the above measures on a more modest scale than we have considered could still delay epidemic growth sufficiently to allow time for ecosystem adaptation and management, therefore reducing the ecological, economic, and social impacts of disease [48]. Such delay would also ‘buy’ time for the development of chemical and biological control tools. Looking more widely into the benefits of disease management, large-scale control measures in Humboldt County should be designed also with the goal of achieving, or maintaining, forestry and other economic enterprises currently impacted by the presence of *P. ramorum*. Finally, the model suggests that the most viable strategy epidemiologically and economically is to control new, smaller foci through early detection, removal of inoculum, and host protection ahead of the epidemic front. These epidemiologically-based control insights should be linked to an understanding of how the viability of management actions is also shaped by pre-existing factors such as economic, social (e.g., patterns of land ownership, acceptability of specific treatment methods), and legal (e.g., state and federal permitting and environmental compliance) constraints [48], [68].

Our results suggest that it is possible to reduce inoculum and to contain the spread of *P. ramorum*, but also indicate that early and aggressive interventions alone might not achieve eradication of this pathogen. These findings are consistent with the epidemiological patterns observed in the northernmost focus of *P. ramorum* incidence in southwest Oregon, where aggressive treatments have contained but not eradicated the pathogen [41], [48]. A new find of *P. ramorum* in northern California should allow us to test the approaches outlined in this paper. In spring 2010, we detected an additional *P. ramorum* outbreak approximately 100 km north of the Redway infection site (Valachovic et al., unpublished). Although small (∼10 ha), the new site is extensive enough to suggest that it has been active for a number of years, but with a long cryptic-infection period. During fall 2010 and spring 2011 the majority of inoculum producing hosts was removed in and around this site.

Our overall conclusions address several challenges about the management and control of emerging plant pathogens in heterogeneous host populations in natural landscapes. Large- scale dispersal, high local and regional sporulation, and a broad host range produce a host landscape with high connectivity that facilitate rapid and extensive invasion [8]. Our study demonstrates that many management actions are ineffective in achieving their stated goal of limiting pathogen spread, but also suggests that efforts to control emerging plant pathogens should be encouraged. Fragmentation of suitable habitat through disturbances such as logging, wildfire, disease, or disease control efforts, may lead to aggregated host distributions. Understanding how landscape structure influences invading species is critical to identifying appropriate management actions to reduce their impacts [4]. At the scale of the Humboldt study area (Fig. 2), the variation in host communities is not sufficient to limit the spread of *P. ramorum* in the long term. However, larger distances between patches of suitable habitat can reduce the likelihood of establishment of invasive species [8]. For example, across California there are regional variations in host availability and weather conditions responsible for spatial refugia, that could remain infection-free for many years despite the potential long-distance dispersal of *P. ramorum* [32]. In addition, disease management actions against emerging pathogens in Humboldt County and elsewhere are likely to be applied unevenly across the landscape because individual landowners assign different value to their forest resources. Further research is needed to understand how the spatiotemporal variation introduced by social dynamics would affect the impact of management treatments and pathogen spread. In other forests worldwide, where environmental conditions are less suitable for *P. ramorum* and related pathogens, the spatial arrangement of treatments could be particularly influential on the efficacy and cost effectiveness of pathogen management. We hope to have shown in this paper that the adoption of informed control measures is, at least, more likely to ameliorate local economic, ecological, and social impacts of disease, while making rational use of limited resources. Moreover, by linking disease control with management practices, it may even be possible to convert challenges into opportunities for shaping ecosystem composition and function for the benefit of communities and the environment.

## Supporting Information

Text S1 We provide further detail on model assumptions and formulation, estimation methods, and predictions.
(PDF)
