# Supplementary material for: Landscape Epidemiology and Control of Pathogens with Cryptic and Long-Distance Dispersal: Sudden Oak Death in Northern Californian Forests
Source: PLoS Comput Biol. 2012 Jan 5;8(1):e1002328. doi: 10.1371/journal.pcbi.1002328 (PMC3252276; doi:10.1371/journal.pcbi.1002328)
Supplement: Text S1 — We provide further detail on model assumptions and formulation, estimation methods, and predictions. (PDF) [file pcbi.1002328.s001.pdf]

Landscape Epidemiology and Control of Pathogens with Cryptic and Long-Distance Dispersal: Sudden Oak Death in Northern Californian Forests

João A N Filipe[1], Richard C Cobb[2], Ross K Meentemeyer[3], Christopher A Lee[4], Yana S Valachovic[4], Alex R Cook[5], David M Rizzo[2], Christopher A Gilligan[1]
[1] Department of Plant Sciences, University of Cambridge, Cambridge, CB2 3EA, UK
[2] Department of Plant Pathology, University of California at Davis, Davis, CA, 95616, USA
[3] Department of Geography and Earth Sciences, University of North Carolina, Charlotte, NC, 28223, USA
[4] University of California Cooperative Extension, Eureka, CA, 95503 USA
[5] Statistics & Applied Probability, National University of Singapore, Singapore
Corresponding author: jf263@cam.ac.uk

Supplementary Information

Development and parameterisation of a model of the transmission of *Phytophthora ramorum* in the landscape (Sec. 1, 2, 4) and additional results from the model (Sec. 3)

1 - Assumptions of the model

We describe in the following paragraphs the main assumptions made in the development of an epidemiological model of the transmission of *Phytophthora ramorum* in the spatiotemporal landscape defined by host and weather variables. The model is at core similar to that in [1] but with differences in formulation and detail as it was designed for a different purpose, namely, predicting regional (as opposed to state-wide) natural spread, and predicting the outcome of control scenarios.

Host landscape: We consider the host landscape comprising redwood-tanoak and Douglas-fir-tanoak forests in Humboldt County, northern California, USA. The spatial resolution of the model is a unit cell (or site, or forest stand) of 250m x 250m, chosen as a compromise between useful amount of information and tractability. Cells are arranged on a square grid. The spatial distribution of known hosts of *P. ramorum* [1,2] is accounted for using a *relative host index, h_i*. This approach allows us to quantify the susceptibility of each non-infected cell to become infected and the suitability of each infected cell to produce infectious spores of the pathogen. The *absolute host index* of each cell i, H_i, was calculated using data on local composition and density of host species (estimated from the CALVEG plant communities database [3] implemented in a Geographic Information System), and a measure of relative susceptibility and infectivity of each host [4]. The index comprises the key known infectious hosts, such as bay laurel (*Umbellularia californica*) and tanoak (*Notholithocarpus densiflorus*), but not lethally-infected non-sporulating hosts, such as coast live oak (*Quercus agrifolia*) and other oak species. The relative index is obtained through division of the absolute index by its spatial mean over the study area ($\bar{H}$),

$$h_i = H_i / \bar{H}\,, \qquad \text{(A1)}$$

and is incorporated in the model by multiplying the transmission rate β (equation A.2). The relative index allows us to compare the transmission rate β against homogeneous landscape conditions (where h_i=1), and to interpret β as the rate of secondary infection of typical cells by a single infected typical cell in a non-infected landscape (see definition of dispersal kernel below).

Biology of the pathogen and host populations: Assumptions and parameter values relative to the lifecycle of *P. ramorum* and to the host population are summarized in Table S1. The dispersal of *P. ramorum* is described by a probability function of the distance between source and target (dispersal kernel) that we assume to be isotropic at the scale of the study area. This working hypothesis is unlikely to be fully accurate at large scales, but we use it for simplicity given our limited data and understanding of the dispersal of the pathogen. We fitted the epidemiological model to the observations using different candidate dispersal kernels and found that a power-law function describes the data much better than a negative exponential (see below). In addition, we extrapolate the best-fit dispersal kernel from the survey area to conditions throughout the wider area to which we apply the model to forecast current and future cryptic infection. This assumption is justified by the pattern of the local landscape and the limited epidemiological data. For simplicity and lack of data we do not explicitly account for topographic variables and features, such as cliffs and valleys, which could potentially affect pathogen dispersal but would be difficult to model.

Epidemiology: 1) Once infection occurs in a unit cell, it spreads and intensifies within the cell, but we only account for presence or absence of infection in each cell. This simplification ignores a transient effect, and amounts to considering an effective level of inoculum that is reached rapidly (but is below the maximum sporulating capacity of the cell), and which we use for calculating the force of infection exerted on other cells. Improving this approximation would require a much larger computational effort in the parameter estimation procedure. However, we believe the approximation will not have affected significantly the model predictions for this system. **2)** We assume that infected cells become immediately infectious, as there is evidence that the latent period of *P. ramorum* is small across its host range [2,5]. **3)** The duration of *P. ramorum* infections is not known, but it is known to be long and host specific (e.g., some hosts die). In the key sporulating host, bay laurel, infected leaves are much more likely to be cast compared with uninfected leaves, which suggests a mechanism by which non-lethal hosts could recover from infection [6], e.g., transmission of infection within a host canopy could cease to be sustained under particularly-long dry periods such as multiple-year droughts. In addition, in stands where tanoak is the only sporulating host all hosts may become infected and be killed by disease; subsequently, some of the dead tanoak would sprout and it is possible that some would become infected and sustain the pathogen; but if sprouting occurred during a long dry period the pathogen would be unlikely to persist and the tanoak stand could recover from infection. Hence, we hypothesize that infections are long but not indefinite (in the absence of external inoculum) because there is no evidence to distinguish between the two possibilities. We allow for long-term interruption in infection by assuming a finite infectious period longer than the time since we started monitoring *P. ramorum* in California [7]. We call the process at the end of the infectious period *recovery* from infection. **4)** To be consistent with the aerial survey data used to parameterize the model, we assume that infected cells become symptomatic upon the emergence of at least one dead tanoak tree in the cell. For simplicity, we also assume that the level of infectivity and the infectious period of an infected cell are, on average, unaffected by the emergence of symptoms. One reason to expect that the infectious period of a cell might be unaffected by the emergence of symptoms is that dead tanoaks re-sprout rapidly and can be re-infected by other sporulating hosts that sustain infection within the stand [7]; host re-sprouting is a key mechanism of host and pathogen persistence in simple stand-level models for this disease (Cobb and Filipe, *unpublished data*).

Humboldt outbreak conditions: We assume there is no significant external inoculum in Humboldt other than the rare (most likely human-mediated) inoculation that initiated the

epidemic outbreak. Our analysis suggests that the epidemic started in 2001 at a site near Briceland/Garberville, 2-3km south of Redway. These values are the arithmetic mean and mode, respectively, of the posterior distributions for the time (between 2000 and 2003) and location (around Redway) of the first cell infected obtained using data-augmented Bayesian MCMC inference. Given that there is no significant impact of the small-scale historic treatments implemented in Humboldt since the outbreak was first reported in 2002 [8] we do not model the effect of these treatments.

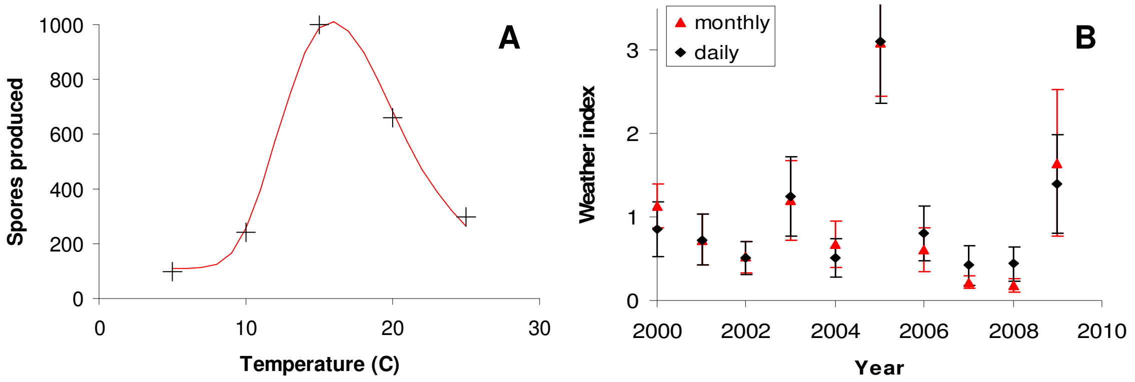

Figure S1 – **A)** The relationship between mean number of zoospores produced by a host infected with *P. ramorum* and temperature [5] and the fitted lognormal function (R^2=0.9959): $f(T) = 108.6 + 904.8\exp(-0.5[\ln(T/15.87)/0.2422]^2)$. **B)** Weather index and 95% confidence intervals calculated using temperature and precipitation datasets from two weather stations in different locations near Redway, Humboldt, CA, one comprising daily records and the other comprising monthly records. There is good agreement between indices based on data from different locations and with different temporal resolution.

Weather: We use a weather index to account for the effect of weather conditions on the probability of non-infected hosts becoming infected and infected hosts sporulating and spreading the pathogen. This is clearly important, as the level of disease detected in aerial surveys and the number of infected hosts detected in sparse ground surveys are highly variable and correlate with weather conditions [4,5,6]. The best predictor for annual increase in *P. ramorum* infection in redwood-tanoak forests in California is thought to be the annual spring rainfall, but temperature is also important [6]. Thus, we define an *annual weather index*, $w(t)$, that multiplies the baseline transmission rate, β_0, to produce a time dependent transmission rate,

$$\beta(t) = w(t)\beta_0 \qquad \text{(A2)}$$

where β_0 is estimated by us. The *basic* weather index for year t, $W(t)$, comprises the joint cumulative effect of rainfall and temperature between April and June of each year, and is calculated as follows:

$$W(t) = \sum_{d \in \{Apr(t),\ldots,Jun(t)\}} r_5(d) f(T(d)) \qquad \text{(A3)}$$

where $r_5(d)$ is the cumulative rainfall over five days up to day d, $T(d)$ is the average daily temperature on day d, and $f(T(d))$ is a measure of the rate of sporulation under favourable moisture (cumulative rainfall) conditions. The accumulation of rainfall over five days is based on results from several field studies showing that transmission events are associated with periods of continuous rainfall, i.e., greater sporulation and infection occur in higher moisture conditions [9,10]. Observations of sporulating hosts [5] exhibit a temperature range within which sporulation can occur, and were well fitted by a lognormal function of temperature

(Fig. S1A). The *actual* weather index, *w(t),* is normalized by the mean ($\bar{W}$) over the decade of 2000-2009,

$$w(t) = W(t) / \bar{W}. \qquad \text{(A4)}$$

This normalization follows the same spirit as that for the host index: it is such that β_0 can be interpreted as the annual transmission rate under average (or under constant) weather conditions. We verified that the weather pattern was consistent among available stations around Redway (Fig. S1B), and thus adopted annually-varying but spatially-uniform weather conditions across the study area. The above model (A2)-(A4) for the effect of weather on pathogen transmission was integrated into the epidemiological model used both for parameter estimation and for prediction. The parameter estimation involved implementing time-dependent hazards and cumulative hazards of infection, which is a computationally intensive task in spatiotemporal data-augmented Bayesian MCMC inference. We also compared variations on the definition of the weather index against disease incidence data and tested the sensitivity of the parameter estimates and model outcomes to these variations, and found (A3) to be a plausible model.

Control and management strategies:

1. Monitoring & removal of inoculum. Monitoring and removal rounds take place synchronously across a control area, with a frequency of 8-12 months, and each visit lasts for about 3 weeks. In order to increase the feasibility of large-scale control, we assume that monitoring and detection are done visually through aerial surveying followed by ground inspection; so infection in a cell can only be detected if there are symptoms visible from the air. Not all cells are inspected, but more effort is spent searching for infection in cells with higher host index, i.e. that are more susceptible and have more bay laurel and/or tanoak. The combined efficacy of detection (which determines the coverage of tree removal) and of removal of inoculum (through tree removal upon detection) is assumed to be 80%. Once an infected cell has been detected its hosts are removed and hosts in adjacent cells are removed pre-emptively. However, tree removal does not have a permanent effect: stands where hosts have been removed can be re-colonized by hosts via re-invasion or re-sprouting within 3-4 years on average. For simplicity, we assume that the vegetation composition and thus the host index are re-instated upon re-colonization, which is a plausible assumption within the time scale of the study. We consider alternative specifications for the control area: two contrasting locations, “at the origin” or “ahead of the origin” (Fig. 2 in the Manuscript), and two possible dimensions, 15km east-west and either 24km or 28km north-south.

2. Host protection using spraying. Host protection is effected through aerial spraying on a large scale of a phosphate-based compound that reduces host susceptibility. AgriFos® is an example of a phosphate that has been applied for this purpose and is efficacious in reducing oak and tanoak susceptibility in field trials [11,12]. Experimentation with aerial application is underway in small experimental plots in Oregon, USA [13], but the long-term efficacy of aerial AgriFos® treatments has not yet been established. In addition, there would be considerable practical obstacles to the implementation of this aerial treatment in California. Therefore, we explore this control option as currently hypothetical but possible in the future. We assume that rounds of aerial spraying take place synchronously across a control area, with the same frequency and duration of application as removal of inoculum. We also assume, based on the results of experimental observation [11,13] that the combined efficacy of coverage and protection is 80% and protection lasts 2 years on average. Although the experiments show a range of host responses to treatment and the latter values are best-response values, we attempt to demonstrate the maximum impact that this form of treatment could achieve if applied. Most phosphates, including AgriFos®, have a preventive effect and cannot be used to cure infection [12]. Moreover, the phosphates are effective in hosts that

develop lethal cankers, but are not effective in preventing foliar infection in bay laurel. Hence, this treatment would target mainly the epidemiological role of tanoak. We consider spraying only in control areas "ahead of the origin", considerably north of Redway. In these areas bay laurel density is low and tanoak dominates, so we assume for simplicity that the potential presence of some bay laurel would not be enough to reduce the overall efficacy of host protection provided by this treatment. Moreover, human-population density is lower north of Redway, minimizing the overlap between human habitation and spraying. Spraying is applied only as part of a mixed strategy that combines protection of non-infected areas "ahead of the origin" with curative treatment (removal) "at the origin" where the pathogen is already established.

3. Host-free zone ("barrier") for containment. An area stretching from east to west across the landscape, with a width of either 5km or 10km, is cleared of all hosts and prevented from being re-colonized through repeated clearing or use of herbicides that prevent re-sprouting. The barrier is situated at the northern edge of the control area "ahead of the origin" (Fig. 1). This measure is implemented in order to contain northward spread of the pathogen. Similar barriers have been under construction just north of the Van Duzen river [14].

2 - Mathematical formulation of the model

In the following sections we provide a mathematical formulation and quantitative assumptions of the epidemiological model for the transmission of *P. ramorum*.

Basic model

We model the spread of *P. ramorum* in the host and weather spatiotemporal landscape as a dynamic process on a meta-population comprising N contiguous subpopulations represented by cells (sites) arranged on a square lattice. Each cell mimics either a forest stand with its own vegetation composition or a patch without vegetation. Cells can be in one of the following states (Fig. S2): *Susceptible* (S), *Infected* (infectious but cryptic) (I), *Diseased* (infectious and symptomatic) (D), *Removed* or culled (R).

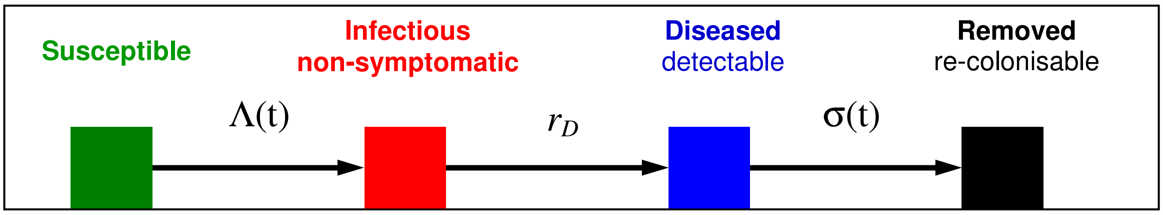

Figure S2 – Compartmental structure of the epidemiological model (see equations (A5)- (A7)).

Natural dynamics. A susceptible cell i can become cryptically infected subject to a force of infection $\Lambda_i(t)$ (equation (A6)) and once infected it can become diseased at rate r_D. Infected sites remain infectious for a very long period, recovering from infection at a very small rate μ. Diseased cells, despite containing dead hosts have the same transmission rate, i.e. are as infectious, and recover at the same rate as cryptically infected cells. When there is control and a cell i becomes removed (through tree removal at rate $\sigma(t)$, see below), it is subsequently re-colonized by susceptible hosts at rate $r_C H_i$ leading to reestablishment of the original host composition and host index (with $H_i \in [0,1]$ the host index, and r_C the re-colonization rate at maximum host availability).

Control dynamics. Control measures are implemented as a sequence of pulses starting at time t_s (corresponding either to year 2010 or 2005) after the estimated time of onset of the outbreak (see below).
(1) Removal of inoculum: a diseased site *i* that is in the control area (Fig. 2 in the Manuscript) can be detected and removed in each intervention round, which is a pulse that occurs with frequency σ_0, lasts ~20 days, and has efficacy ε_R ~ 0.8 (ε_R is the probability of detection and successful tree removal of a site during one round if the site were independent of other sites and not re-exposed). We adopt a *host-targeted optimized removal strategy* where the likelihood of surveying and detecting disease in a site within the control area is proportional to the site's relative host-index – we are assuming that a cell with high host-index is likely to be at greater risk of epidemic spread to other cells and of damage to its own vegetation.
(2) Ring removal: when a diseased site is removed, sites within a radius r_R of the centre of the diseased site are also removed if not already removed (regardless of their status being susceptible, infected, or diseased); we adopt r_R ~1.5 cell size, hence ring removal targets the 8 adjacent cells around the central cell (a Moore neigbouhood).
(3) Spraying with a protective phosphate: Spraying is applied with the same frequency and has the same efficacy as removal; but it is applied exclusively in the control area "ahead of the origin", which may or not be ahead of the epidemic front.
(4) Host-free barrier: all sites in this area are non-susceptible (removed) after time t_s.

Parameter	Description	Value	Source
**Life cycles of pathogen & host population**			
Time to symptoms, $1/r_D$	Mean time between *P. ramorum* infection in the stand and tanoak mortality, inverse of disease-induced mortality rate	2.5 yr [2.32, 2.89]	MCMC estimation [95% credible interval]
Infectious period, $1/\mu$	Mean duration of *P. ramorum* infection in a stand	10 yr	See main text; greater than the duration of isolated outbreaks
r_D	Rate of acquisition of detectable symptoms (rate of tanoak mortality)	0.4 yr^{-1} [0.35, 0.43]	MCMC estimation [95% credible interval]
μ	Rate of recovery from infection	1/10 yr^{-1}	See main text
r_C	Rate of re-colonization by hosts upon host removal	0.25-0.35 yr^{-1}	[7] and natural response to woodland management (Valachovic, unpublished data)
**Transmission**			
Baseline transmission, β_0	Mean rate at which an infected cell infects another cell	1.8 yr^{-1} [1.67, 1.99]	MCMC estimation [95% credible interval]
α	Exponent of power-law dispersal kernel	3.55 [3.41, 3.70]	MCMC estimation [95% credible interval]
**Control: treatment via removal**			
σ_0	Frequency of follow-up of treatment in the control area	8-12 mns for 3 weeks	Assumed
Duration, $1/r_C$	Mean time for hosts to re-colonize or re-sprout in a stand after curative or pre-emptive tree removal	3-4 yr	See r_C above
Efficacy, ε_R	Combined efficacy of detection and removal of host and inoculum	80%	Assumed; matches host protection treatment
r_R	Radius of removal about centre of the cell where disease is detected	1.5 cell unit (375 m)	Assumed

Control: protection via aerial spraying			
σ_0	Frequency of follow-up of treatment in the control area	8-12 mns	Assumed
Duration, d_p	Mean duration of protection	2 yr	[11]
Efficacy, ε_P	Combined efficacy of phosphate deposition and protection	80%	[11]

Table S1 – Main parameters of the epidemiological model.

Predictive model

In order to make forward predictions regarding pathogen spread under natural dynamics or under different control scenarios we used the following spatially-explicit, continuous-time probabilistic formulation of the basic model described above. The model shares features with spatially-structured metapopulation models [15]. The probabilities that cell i is in each of the possible states, Susceptible, Infected**,** Diseased, or (if there is control) Removed, i.e., $P_{i,S}$, $P_{i,I}$, $P_{i,D}$, and $P_{i,R}$, respectively, are governed by the system of differential equations:

$$\begin{aligned}
\frac{dP_{i,S}}{dt} &= \mu\left[P_{i,I}+P_{i,D}\right]+r_C H_i P_{i,R}-\left[\Lambda_i(t)+\sigma_{i,n}(t)\right]P_{i,S}\\
\frac{dP_{i,I}}{dt} &= \Lambda_i(t)P_{i,S}-\left[\mu+r_D+\sigma_{i,n}(t)\right]P_{i,I}\\
\frac{dP_{i,D}}{dt} &= r_D P_{i,I}-\left[\mu+\sigma_{i,n}(t)+\sigma_{i,D}(t)\right]P_{i,D}\\
\frac{dP_{i,R}}{dt} &= \left[\sigma_{i,n}(t)\left[P_{i,S}+P_{i,I}+P_{i,D}\right]+\sigma_{i,D}(t)P_{i,D}\right]-r_C H_i P_{i,R}
\end{aligned} \qquad \text{(A5)}$$

The initial conditions, at the estimated time of onset of the outbreak, are $P_{i,S}=1, P_{i,I}=0, P_{i,D}=0, P_{i,R}=0$, except at the cell estimated to be the location of the first infection, where $P_{i,S}=0, P_{i,I}=1, P_{i,D}=0, P_{i,R}=0$. The force of infection is given by:

$$\Lambda_i(t)=\beta(t)\sum_{j\neq i}A_j B_i(t)C_{j,I+D;i,S}K(d_{ij}) \ , \qquad \text{(A6)}$$

where $\beta(t)=\beta_0 w(t)$ is the transmission rate, with $w(t)$ an annual index of weather fluctuation about a 10 year average (equations (A2)-(A4)) and β_0 the baseline rate; $K(d_{ij})$ is the dispersal kernel (see below); $A_j=h_j$ is the infectivity of donor site j, and $B_i(t)=h_i[1-p_i(t)]$ is the susceptibility of receptor site i, with $p_i(t)$ the level of host protection in cell i at time t; $C_{j,I+D;i,S}$ is the conditional probability that site j is infectious (with cryptic or symptomatic infection) given that site i is susceptible. To first order of approximation, we assume that $C_{j,I+D;i,S}\approx P_{j,I}+P_{j,D}$, which we expect to be a reasonable approximation to the infection pattern [16] given that dispersal is not very localized, as indicated by the estimated exponent of the power-law dispersal kernel (see below). The rates of removal in diseased cells and in non-diseased cells (through radial removal) are given, respectively, by:

$$\begin{aligned}
\sigma_{i,D}(t) &= \sigma(t)h_i\delta_i\\
\sigma_{i,n}(t) &= \sum_{j\in\mathbb{Z}_i}\sigma(t)h_j\delta_j P_{j,D} \ ,
\end{aligned} \qquad \text{(A7)}$$

where $\delta_i=1$ if cell i is in the control area where *removal* takes place and $\delta_i=0$ otherwise; $\mathbb{Z}_i$ is the set of cells adjacent to cell i. The time-dependent function $\sigma(t)$ is the baseline rate of treatment, with form: $\sigma(t)=\text{amplitude}\times\exp[-\Delta t/\text{duration}]$, where $\Delta t=t-T(t)$ is the time

elapsed since the start of the current pulse (or round), and the amplitude is calculated such that a treatment round has duration of ~3 weeks (after which σ~*0*), and efficacy ε_R and frequency σ_0 as in Table S1. The effect of spaying in cell *i* is represented by a time-decaying level of protection $p_i(t) = \delta_i \varepsilon_p [1 + 2\Delta t / d_p] \exp[-2\Delta t / d_p]$, corresponding to a gamma-distributed duration with mean d_p, and where $\delta_i = 1$ if cell *i* is in the control area where *spraying* takes place and $\delta_i = 0$ otherwise.

Dispersal kernel: We considered as candidates for the probability that pathogen spores disperse a distance *d* from the source, power-law and negative exponential functional forms [17], with generic form:

$$\begin{aligned} K_P(d) &= C_1 / d^{\alpha} \\ K_E(d) &= C_2 \exp[-d/\alpha] \end{aligned}, \qquad \text{(A8)}$$

where the constants C_1 and C_2 are such that the functions are normalized to 1 on the plane, excluding the area of the source cell. The latter condition means that we are only considering dispersal events where pathogen spores produced in a source cell are deposited in a different cell within the study area or further beyond, i.e. transmission in (A6) is conditional on spores being dispersed outside the source cell. The rationale for this choice is that we do not keep track of the infection process within a cell, which is below the resolution of the observations. In addition, we use an effective kernel that results from integrating the point kernel (A8) over the area of the target cell, accounting for all possible ways through which the target cell can become infected by a given source cell. By fitting the epidemiological model to the spatiotemporal observations (using the Bayesian MCMC approach described below) we found that a power-law fitted the data significantly better than a negative-exponential (Deviance Information Criterion = 10691 and 13309, respectively [18]). The superiority of the power-law kernel is also evident from visual comparison of the predicted and observed patterns of disease, which are dispersive in nature (Fig. S3), and from inspection of the traces of the likelihood (A10). This result suggests that *P. ramorum* can disperse over large distances with a long tail of low probability.

Probability of invasion: We calculated the probability of invasion at given time of a given area, Ω, representing for example a protected at-risk area, as

$$P_{inv}(\Omega) = 1 - \prod_{i \in \Omega} P_{i,S} \,. \qquad \text{(A9)}$$

We used this definition to define the probability of invasion of the 'Target area' under protection (Fig. 2 in the Manuscript) and, by using narrow areas at successive distances from the focus, to quantify the progress of the epidemic front as shown in Fig. 3 in the Manuscript.

3 - Results

The dispersal kernel is a component of the epidemiological model that can greatly influence the model's ability to predict the spread of the pathogen and the impact of management strategies. We contrasted the goodness-of-fit of the epidemiological models based on each of the dispersal kernel forms through a visual comparison of the predicted spatial pattern (Fig. S3) and temporal progress (Fig. S4) of disease against the survey data. Clearly, the power-law kernel not only fits the data much better in relation to the exponential kernel, but it also fits the data well in absolute terms. In addition, we found that local control is effective in sustaining a low local level of infection (Fig. S5).

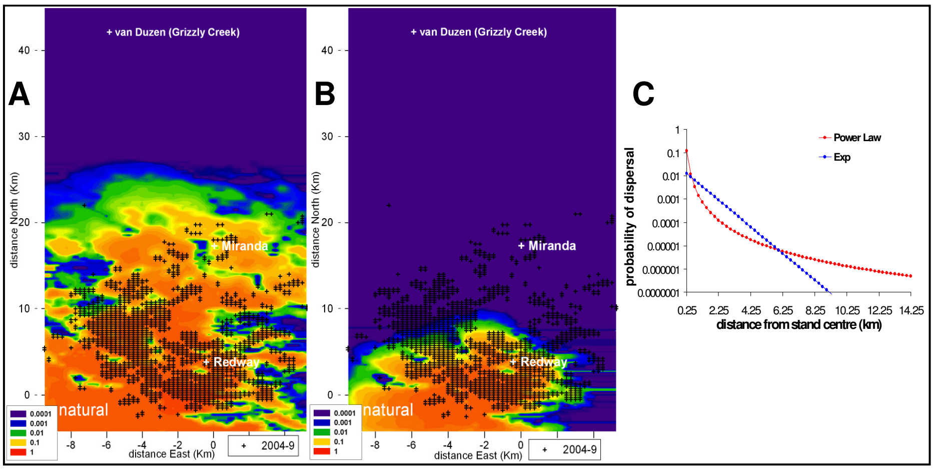

Figure S3 – Goodness-of-fit of the model: spatial distribution of disease in 2009. Model with alternative dispersal kernels: A) power-law, B) negative exponential. Comparison of the predicted probability of presence of disease (on log scale) in the absence of management with the cumulative distribution of disease (tanoak mortality) caused by *P. ramorum* estimated from the Humboldt survey data (2004-2009). C) Dispersal kernel functions corresponding to A and B.

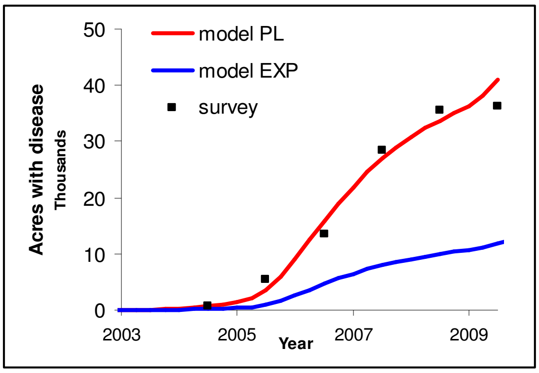

Figure S4 – Goodness-of-fit of the model: temporal increase in the area with disease. Comparison of the area with disease (tanoak mortality) caused by *P. ramorum* estimated from the Humboldt survey data (2004-2009) and as predicted by the model in the absence of management fitted with alternative dispersal kernels: power-law (PL) and negative exponential (EXP).

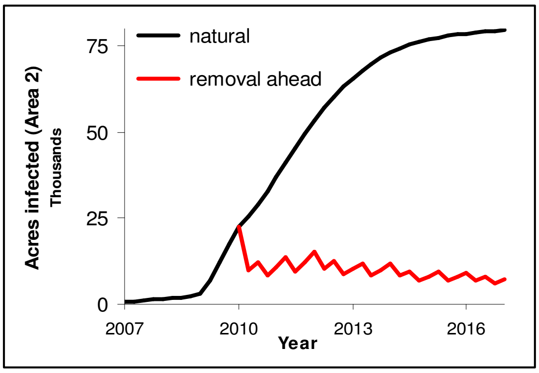

Figure S5 – Demonstration of local effectiveness of control. Even when there is no outbreak containment because the control area is <u>smaller</u> than the infection area (which includes symptomatic and cryptic infections), repeated rounds of removal of symptomatic infection in Area 2 (from 2010) keep infection at a low level. Under sustained control (wriggling red curve) the local basic reproductive number (R_0) is < 1; this means that the epidemic should decline over time; however, continued re-infection through long-distance dispersal from non-controlled areas (case B in Fig. 1 in the Manuscript) prevents that from happening.

We note that it was not possible to cross-validate the model against independent representative data because no such data were available. Indeed, other outbreaks of *P. ramorum* have been disturbed by disease and forest management (e.g., in southern Oregon) and extensive wildfires (e.g., in Big Sur, California), so a model applied to those areas would have to include additional processes to represent the disturbances. Moreover, the natural and urban landscape conditions of other eco-regions where outbreaks have occurred are significantly different from those of our study area of Humboldt County. While it is possible to adapt the model for some of the changes in landscape variables, that would have involved additional assumptions and blurred the outcome of cross-validation attempts, and, as we say in the Manuscript, it is a priority to predict the impact of potential management strategies in Humboldt County. Moreover, it would not have been meaningful to subdivide our dataset because of heterogeneities of pathogen spread in space and time (c.f., “Results - Predicted natural spread” in the Manuscript).

Range of scenarios for pathogen spread. We considered three scenarios representing a likely range of ability or potential of the pathogen to spread in the host landscape: “high”, “medium” and “low” pathogen spread scenarios. The medium spread case corresponds to the median values of the marginal posterior distribution of the estimated parameters characterizing pathogen transmission and period of cryptic infection (Table S1 and Fig. S7). We used these values in all results presented except in Fig. S6. We defined the “high-spread” (“low-spread”) scenario using a combination of parameter values that leads to greater (smaller) potential of the pathogen to spread and to lesser (greater) potential for detection of symptoms after infection; these parameter values correspond to the percentiles associated with the 95% credible regions of the marginal posterior distributions (Fig. S7). The parameter values for the high, medium, and low spread scenarios are: (α, β, r_C) = (3.70, 1.67, 0.43), (3.55, 1.80, 0.40), and (3.41, 1.99, 0.35), respectively. We expect the pathogen spread potential to be inversely related to the efficacy of treatments. Considering epidemic progress in the absence of treatment and the impact of different treatments over the range of pathogen-spread scenarios, allows us to assess the degree to which the results for the medium-spread scenario are representative of the system’s behaviour and response to treatment or might change under uncertainty about parameters or under condition presented by other host-pathogen systems. The growth in epidemic size over time, the delay in invasion of yet uninfected areas, and the ranking in the relative impact of the different control strategies are largely in accord with our expectations (Fig. S6).

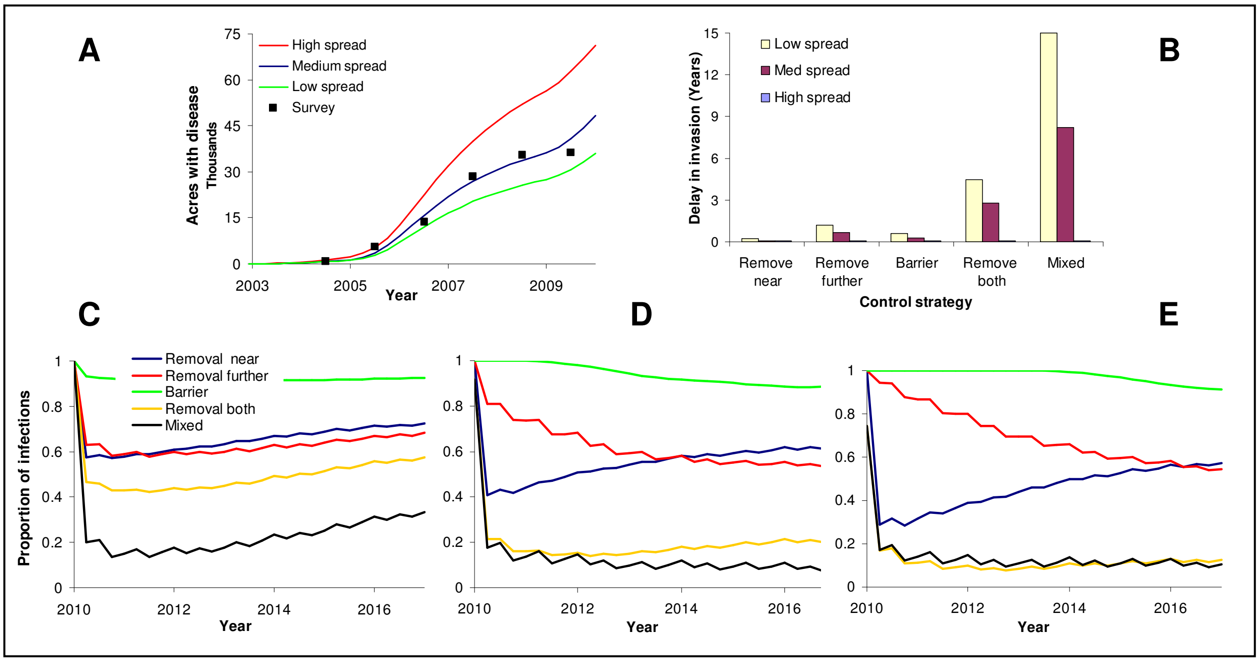

Figure S6 – Scenarios for pathogen spread and effectiveness of treatments. Parameter scenarios with "high", "medium", and "low" pathogen spread, corresponding to low, medium, and high effectiveness of treatment. **A)** Predicted epidemic progress in the absence of pathogen control. **B)** Delay in pathogen invasion of the protected area (Area 3, Fig. 1 in the Manuscript) resulting from implementation of each control strategy starting in 2010 (Fig. 4 and 5 in the Manuscript); delays in the "high spread" scenario are nearly zero because the protected area is already invaded by 2010. **C-E)** Predicted epidemic progress in each control strategy, represented by the proportion of the infections that occur with treatment relative to those that occur without treatment, for the high, medium, and low spread scenarios (C, D, E, respectively).

4 - Parameterization of the model using Bayesian MCMC inference

We parameterized the natural dynamics of the predictive model (namely the transmission, dispersal, and mortality parameters) using Bayesian Markov chain Monte Carlo inference methods [19,20] with data augmentation and reversible jump [21,22] applied to incomplete and censored spatiotemporal observations [23,24]. For inference we used a spatially-explicit, stochastic and continuous-time formulation of the predictive model. We make the simplification of not considering the very-slow-rate process of recovery of infection which amounts to using a SI (Susceptible-Infected) rather than a SIS compartmental model. This step greatly facilitates implementation of the data-augmented component of the MCMC algorithm that deals with missing data, which would otherwise be much more complex and computationally expensive. We justify the approximation on the basis that the time window of observations (5yrs) is smaller than the assumed mean duration of infections Table S1); as a result, any MCMC proposal to reverse the status of an infected site would be very likely to be rejected.

Conditioning on the unobserved times during the survey period, of infection and symptoms (mortality), or of infection only, the likelihood function can be calculated directly (adopting suitable hazard functions, φ_i and Φ_m, for infection and symptoms):

$$
\begin{aligned}
&L\left(\text{parameters} \mid \text{uncensored data}\right) = \\
&\prod_{s\,\in\,\{\text{sites never infected}\}} \exp\left\{-\int_{T_{\min}}^{T_{\max}} \varphi_s(u)du\right\} \\
&\prod_{\substack{i\,\in\,\{\text{sites ever infected}\\ \text{but not observed}\}}} \varphi_i(t_i)\exp\left\{-\int_{T_{\min}}^{t_i} \varphi_i(u)du\right\}\exp\left\{-\int_{t_i}^{T_{\max}} \Phi_m(u)du\right\} \qquad \text{(A10)} \\
&\prod_{\substack{m\,\in\,\{\text{sites ever}\\ \text{symptomatic}\}}} \varphi_i(t_{m_1})\exp\left\{-\int_{T_{\min}}^{t_{m_1}} \varphi_i(u)du\right\}\Phi_m(t_{m_2} \mid t_{m_1})\exp\left\{-\int_{t_{m_1}}^{t_{m_2}} \Phi_m(u)du\right\}
\end{aligned}
$$

where 'ever' and 'never' refer to the time window of the surveys. We augmented the parameter vector to include these unobserved event times and used MCMC sampling to integrate over values of these times that are consistent with the observed (censored) times [25]. We then applied the Metropolis–Hastings algorithm for the acceptance of proposals for the main parameters, the unobserved event times, and the reversal of previously accepted infections that did not lead to observed disease in the surveys. We used independent, flat, non-informative prior distributions for the parameters, and made joint proposals for the main parameters using pre-sampled covariance matrices. We tuned the variances in the proposal distributions in order to reach asymptotic acceptance rates expected to yield optimal mixing, and run the chain over 100,000 iterations after an appropriate burn-in period. This inferential approach allowed us to select among candidate dispersal kernels (Fig. S3 and Section 2), and to sample posterior densities (Table S1 and Fig. S7) for the main parameters and for the time and location of the first site being infected.

To test convergence and mixing of the chain we utilized several indicators and diagnostics, including: visual assessment of the likelihood trace and stabilization of the acceptance rates of the main parameters; autocorrelation function of the main parameter traces; and, shape of the marginal posterior distributions of the main parameters and their invariance to different start values (Fig. S7).

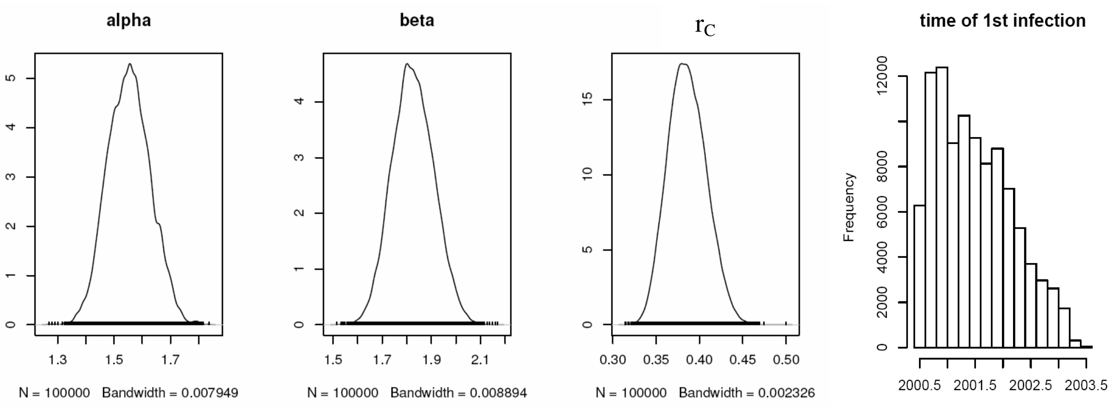

Figure S7 – Marginal posterior densities of the main parameters and time of first infection.

References

1. Meentemeyer RK, Cunniffe NJ, Cook AR, Filipe JAN, Hunter RD, et al. (2011) Epidemiological modeling of invasion in heterogeneous landscapes: Spread of sudden oak death in California (1990–2030). Ecosphere 2: art17.
2. Rizzo DM, Garbelotto M, Hansen EA (2005) *Phytophthora ramorum*: Integrative research and management of an emerging pathogen in California and Oregon forests. Annu Rev Phytopathol 43: 309-335.
3. USDA (2003) Forest Service RSL, CALVEG Vegetation Mapping Program. pp. Sacramento, California, USA.
4. Meentemeyer R, Rizzo D, Mark W, Lotz E (2004) Mapping the risk of establishment and spread of sudden oak death in California. For Ecol Manage 200: 195-214.
5. Davidson JM, Wickland AC, Patterson HA, Falk KR, Rizzo DM (2005) Transmission of *Phytophthora ramorum* in mixed-evergreen forest in California. Phytopathology 95: 587-596.
6. Davidson JM, Patterson HA, Rizzo DM (2008) Sources of inoculum for *Phytophthora ramorum* in a redwood forest. Phytopathology 98: 860-866.
7. Cobb RC, Meentemeyer RK, Rizzo DM (2010) Apparent competition in canopy trees determined by pathogen transmission rather than susceptibility. Ecology 91: 327-333.
8. Valachovic Y, Lee C, Marshall J, Scanlon H. Wildland Management of *Phytophthora ramorum* in Northern California Forests. In: Frankel SJ, Kliejunas JT, Palmieri KM, editors; 2008 March 2007; Santa Rosa, California. Pacific Southwest Research Station, Forest Service, U.S. Department of Agriculture. pp. 305-312.
9. Condeso TE, Meentemeyer RK (2007) Effects of landscape heterogeneity on the emerging forest disease sudden oak death. J Ecol 95: 364-375.
10. Davidson JM, Patterson HA, Wickland AC, Fichtner EJ, Rizzo DM (2011) Forest Type Influences Transmission of Phytophthora ramorum in California Oak Woodlands. Phytopathology 101: 492-501.
11. Garbelotto M, Schmidt DJ (2009) Phosphonate controls sudden oak death pathogen for up to 2 years. Calif Agric 63: 10-17.
12. Garbelotto M, Schmidt DJ, Harnik TY (2007) Phosphite injections and bark application of phosphite + Pentrabark™ control sudden oak death in coast live oak. Arboriculture Urban Forestry 33: 309-317.
13. Kanaskie A, Hansen E, Sutton W, Reeser P, Choquette C. Aerial Application of Agri-Fos to Prevent SOD in Oregon Tanoak Forests. In: Frankel SJ, Kliejunas JT, Palmieri KM, editors; 2010 July 2009; Santa Cruz, California. Pacific Southwest Research Station, Forest Service, U.S. Department of Agriculture. pp. 225-232.
14. Cannon P (2008) Van Duzen No Host Zone. In: COMTF, editor. "Sudden Oak Death: A Decade of Management Challenges", California Oak Mortality Task Force 2008 General Meeting. San Rafael, California, USA.
15. Hanski I, Ovaskainen O (2000) The metapopulation capacity of a fragmented landscape. Nature 404: 755-758.
16. Filipe JAN, Maule MM (2003) Analytical methods for predicting the behaviour of population models with general spatial interactions. Math Biosci 183: 15-35.
17. Minogue KP (1989) Diffusion and Spatial Probability Models for Disease Spread. In: Jeger MJ, editor. Spatial Components of Plant Disease Epidemics. New Jersey: Prentice-Hal. pp. 127-143.
18. Spiegelhalter DJ, Best NG, Carlin BR, van der Linde A (2002) Bayesian measures of model complexity and fit. J R Stat Soc Ser B-Stat Methodol 64: 583-616.

19. Gilks WR, Richardson S, Spiegelhalter DJ (1996) Markov Chain Monte Carlo in Practice. London: Chapman and Hall.
20. Gelman A, Carlin J, Stern H, Rubin D (2004) Bayesian Data Analysis. Boca Raton: Chapman and Hall/CRC.
21. Cappe O, Robert CP, Ryden T (2003) Reversible jump, birth-and-death and more general continuous time Markov chain Monte Carlo samplers. J R Stat Soc Ser B-Stat Methodol 65: 679-700.
22. Cooper BS, Medley GF, Bradley SJ, Scott GM (2008) An augmented data method for the analysis of nosocomial infection data. Am J Epidemiol 168: 548-557.
23. Gibson GJ (1997) Markov chain Monte Carlo methods for fitting spatiotemporal stochastic models in plant epidemiology. Appl Stat-J R Stat Soc 46: 215-233.
24. Gibson GJ, Otten W, Filipe JAN, Cook A, Marion G, et al. (2006) Bayesian estimation for percolation models of disease spread in plant populations. Stat Comput 16: 391-402.
25. Gibson GJ, Renshaw E (1998) Estimating parameters in stochastic compartmental models using Markov chain methods. IMA J Math Appl Med Biol 15: 19-40.
